# The effectiveness of physiologically based early warning or track and trigger systems after triage in adult patients presenting to emergency departments: a systematic review

**DOI:** 10.1186/s12873-017-0148-z

**Published:** 2017-12-06

**Authors:** Francesca Wuytack, Pauline Meskell, Aislinn Conway, Fiona McDaid, Nancy Santesso, Fergal G. Hickey, Paddy Gillespie, Adam J. N. Raymakers, Valerie Smith, Declan Devane

**Affiliations:** 1 School of Nursing & Midwifery, National University of Ireland Galway, Galway, County Galway, Ireland; 2 Health Research Board Trials Methodology Research Network, Galway, Ireland; 3 Nurse Lead, National Emergency Medicine Programme/Clinical Nurse Manager, Emergency Department, Naas General Hospital, Naas, County Kildare Ireland; 4 Department of Health Research Methods, Evidence, and Impact, McMaster University, 1280 Main St. W., HSC-2C15, Hamilton, ON L8S 4K1 Canada; 5 Sligo University Hospital, Sligo, Ireland; 6 Health Economics & Policy Analysis Centre (HEPAC), School of Business & Economics, National University of Ireland Galway, Galway, County Galway, Ireland; 7 Health Economics & Policy Analysis Centre (HEPAC), School of Business & Economics, National University of Ireland Galway, Galway, County Galway, Ireland

**Keywords:** Emergency department, Early warning systems, Systematic review

## Abstract

**Background:**

Changes to physiological parameters precede deterioration of ill patients. Early warning and track and trigger systems (TTS) use routine physiological measurements with pre-specified thresholds to identify deteriorating patients and trigger appropriate and timely escalation of care. Patients presenting to the emergency department (ED) are undiagnosed, undifferentiated and of varying acuity, yet the effectiveness and cost-effectiveness of using early warning systems and TTS in this setting is unclear. We aimed to systematically review the evidence on the use, development/validation, clinical effectiveness and cost-effectiveness of physiologically based early warning systems and TTS for the detection of deterioration in adult patients presenting to EDs.

**Methods:**

We searched for any study design in scientific databases and grey literature resources up to March 2016. Two reviewers independently screened results and conducted quality assessment. One reviewer extracted data with independent verification of 50% by a second reviewer. Only information available in English was included. Due to the heterogeneity of reporting across studies, results were synthesised narratively and in evidence tables.

**Results:**

We identified 6397 citations of which 47 studies and 1 clinical trial registration were included. Although early warning systems are increasingly used in EDs, compliance varies. One non-randomised controlled trial found that using an early warning system in the ED may lead to a change in patient management but may not reduce adverse events; however, this is uncertain, considering the very low quality of evidence. Twenty-eight different early warning systems were developed/validated in 36 studies. There is relatively good evidence on the predictive ability of certain early warning systems on mortality and ICU/hospital admission. No health economic data were identified.

**Conclusions:**

Early warning systems seem to predict adverse outcomes in adult patients of varying acuity presenting to the ED but there is a lack of high quality comparative studies to examine the effect of using early warning systems on patient outcomes. Such studies should include health economics assessments.

**Electronic supplementary material:**

The online version of this article (10.1186/s12873-017-0148-z) contains supplementary material, which is available to authorized users.

## Background

Serious clinical adverse events are related to physiological abnormalities and changes in physiological parameters, such as blood pressure, pulse rate, temperature, respiratory rate, level of consciousness, often precede the deterioration of patients [1–4]. Early intervention may improve patient outcomes and failure to recognise acute deterioration in patients may lead to increased morbidity and mortality [5, 6]. Early warning systems and track and trigger systems (TTS) use routine physiological measurements to generate a score with pre-specified alert thresholds. Their aim is to identify patients at risk of deterioration early and trigger appropriate and timely responses known as escalation of care.

Early warning systems are used increasingly in acute care settings and several countries have developed National Early Warning Scores (NEWS). In Ireland, the National Clinical Guideline on the use of NEWS for adult patients came into effect in 2013 [7]. In the UK, The Royal College of Physicians (RCoP) published a National Early Warning Score in 2012 [8], and the National Institute for Health and Care Excellence (NICE) recommends the use of a TTS to monitor hospital patients [9]. In Australia, the Early Recognition of Deteriorating Patient Program introduced a TTS [10]. Similarly, in the USA, Rapid Response Systems with fixed “Calling Criteria” are recommended to trigger adequate medical response [11].

Many acutely ill patients first present to the emergency department (ED). The ED is a complex environment, distinctly different from other hospital departments. Visits are unscheduled and patients attend with undiagnosed, undifferentiated conditions of varying acuity. Medical staff must care for several patients simultaneously, deal with constantly shifting priorities and respond to multiple demands due to the unpredictable nature of the ED environment [12, 13]. Initial triage determines the priority of patients’ treatments but following triage, continuous monitoring and prompt recognition of deteriorating patients is crucial to escalate care appropriately. Early warning systems are sometimes used as an adjunct to triage for early identification of deterioration in the ED, particularly in situations of crowding [14]. Common early warning systems such as the Modified Early Warning Score (MEWS) [15] are used frequently and validated against specific subgroups of patients (e.g. acute renal failure, myocardial infarction, etc.) but may not be directly transferable to an ED setting [14] where patients present with a variety of unspecified conditions. There was an urgent need to evaluate the use of early warning systems and TTS in the ED.

The review addressed five objectives:

1. To describe the use, including the extent of use, the variety of systems in use, and compliance with systems used, of physiologically based early warning systems or TTS for the detection of deterioration in adult patients presenting to the ED;
2. To evaluate the clinical effectiveness of physiologically based early warning systems or TTS in adult patients presenting to the ED;
3. To describe the development and validation of such systems;
4. To evaluate the cost effectiveness, cost impact and resources involved in such systems;
5. To describe the education programmes, including the evaluation of such programmes, established to train staff in the delivery of such systems.

## Methods

### Study design & scope

We conducted a systematic review, which we report according to the PRISMA guidelines [16]. The scope is presented in Table 1 using the PICOS (Population, Intervention, Comparison, Outcomes, types of Studies) format.

**Table 1:** Study selection criteria

P	Adult patients presenting to the ED following initial triage.
I	Early warning systems or TTS, relying on periodic observation of selected, routinely recorded, physiological parameters, to promptly recognise deteriorating patients and trigger escalation of care based on pre-set response criteria. Condition-specific systems; for example, the Mortality in Emergency Department Sepsis (MEDS) score were excluded from this review. Educational programmes for healthcare professionals concerning such early warning systems or TTS.
C	Non-use of the systems or the use of alternative systems of physiological monitoring. Non-use or use of alternative educational programmes concerning early warning systems or TTS.
O	• Extent of use of early warning systems or TTS • Types of early warning systems or TTS in use • Number and type of clinical guidelines (regional, national, international) related to such systems • Clinical outcomes ▪ Death ▪ Critical illness (collapse – cardiac or respiratory arrest, haemorrhage, sepsis etc.) ▪ Admission to intensive care unit (ICU) ▪ Length of hospital stay (days) • Sensitivity of early warning systems or TTS for adverse outcome/critical illness criterion • Specificity of early warning systems or TTS for adverse outcome/critical illness criterion • Positive predictive value of early warning systems or TTS for adverse outcome/critical illness criterion • Negative predictive value of early warning systems or TTS for adverse outcome/critical illness criterion • Economic measures of healthcare: ▪ Use of healthcare resources associated with early warning systems or TTS use, including direct medical resource costs (staff time, education time and cost, additional referrals), indirect costs (associated with loss of productivity) and other non-medical costs (e.g. patient out-of-pocket expenses) ▪ Cost savings, cost effectiveness measures such as Incremental Cost-Effectiveness Ratios (ICERs), Quality Adjusted Life Years (QALYs) • Types of education programmes • Strategies and methods to evaluate education programmes of early warning systems or TTS
S	The following six types of studies were included: a. *Descriptive studies – types and use of systems*: Studies that described types or variety of early warning systems or TTS used and the extent to which they were used in clinical practice. b. *Descriptive studies – compliance* *:* Studies that described compliance with early warning systems or TTS in clinical practice. c. *Descriptive studies – education programmes* *:* Studies that described education programmes to train healthcare professionals in delivering early warning systems or TTS. d. *Effectiveness studies* *:* Studies that examined the effectiveness of an early warning system or TTS on outcomes for adults admitted to the ED, following triage and that had a controlled design (i.e., RCTs, non-RCTs, controlled before-and-after studies, interrupted time series designs and cohort studies with historical controls). Studies that evaluated the effects of the system on relevant outcomes without control (e.g. case series, cohort studies without historical control) were included in the descriptive category. e. *Development and validation studies* *:* Development studies were defined as studies that focused on the development of early warning systems or TTS while validation studies assessed the predictive ability of such systems. Studies in this category needed to include adult patients both with and without the reference outcome (such as admission to intensive care or mortality) or were otherwise considered a descriptive study. For the purpose of classification, we regarded studies as ‘development’ studies if reference ranges, parameters, and/or design of scoring systems were identified based on the outcomes of the study sample (for example, through the use of receiver operating characteristics [ROC] curves). In validation studies, such reference criteria were already determined and their predictive ability was evaluated in a new sample of patients. f. *Health economics* *:* Full economic evaluation studies (cost-effectiveness analysis, cost-utility analysis and cost-benefit analysis), cost analysis and comparative resource use studies comparing early warning systems or TTS to one or more standard treatments. These may have included any study that met the eligibility criteria for the review of effectiveness; hence studies in other categories might have been also been included here.

### Search strategy

**Fig. 1:**
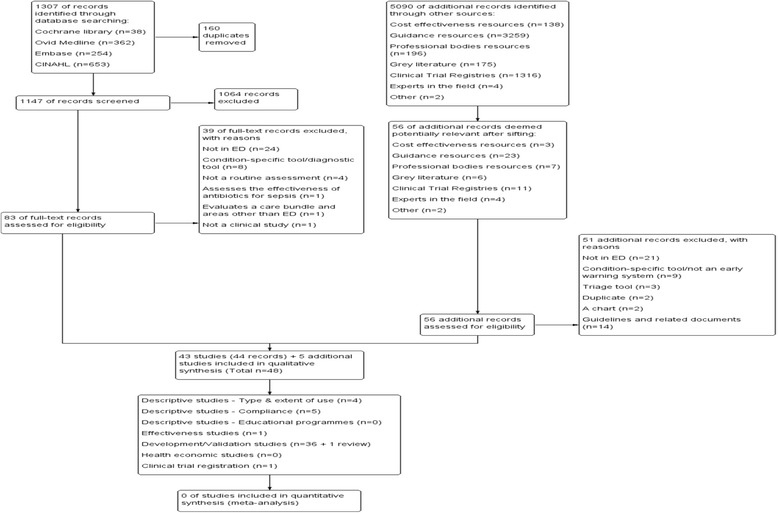
Search and selection Flow diagram. We searched both electronic databases, cost-effectiveness resources, professional bodies’ websites, clinical trial registries and grey literature resources. Experts in the fields were also contacted. We conducted double independent study selection based on title/abstract and full-text

Search strategies using keywords and subject terms were developed for four electronic databases: the Cochrane Library (all databases therein up to 4 March 2016), Ovid Medline (up to 4 March 2016), Embase (up to 22 February 2016) and CINAHL (up to 4 March 2016). Additional grey literature resources that were searched included cost-effectiveness resources (*n* = 4; up to 11 March 2016), guidance resources (*n* = 6; up to 13 March 2016), professional bodies’ resources (*n* = 22; up to 11 March 2016), grey literature resources (*n* = 3; up to 13 March 2016) and clinical trial registries (n = 4; up to 13 March 2016). The searches were not restricted by language, however, only data in English were included. Full details of search strategies are provided in Additional file 1. Details of the search results are presented in Fig. 1 [16].

### Study selection & extraction

Two reviewers (FW, and PM or SD) independently screened the titles/abstracts. For additional resources, the information specialist (AC) sifted through the search results for potentially eligible studies. Full text reports from databases and additional resources were assessed for inclusion by two reviewers independently (FW, PM) and discrepancies were resolved by discussion or by involving a third person (DD).

Data extraction forms were designed for each of the six types of studies. Data extraction was completed by two reviewers (FW, PM). Each reviewer extracted data from half of the included reports and 50% of entries were checked by a second reviewer for accuracy. The data elements that were extracted are available in Additional file 2. Two reviewers (FW, and VS or DD) independently assessed the Risk of Bias (ROB)/methodological quality of the included reports, using the instruments listed in Table 2.

**Table 2:** Instruments used to assess risk of bias and quality of reports

Study design	Risk of bias (ROB)/quality assessment tool^a^
Descriptive studies	Adapted from National Institute of Health checklist [69]
Effectiveness studies – RCTs	Cochrane risk of bias tool [70]
Effectiveness studies – non-RCTs	EPOC quality assessment for quantitative studies [70, 71]
Systematic reviews	AMSTAR
Economic evaluations	British Medical Journal Checklist for authors and peer-reviewers of economic submission [72]; Checklist for quality assessment in economic decision-analytic models [73]
Development and validation studies	Quality Assessment Tool adapted from Kansagara et al. (2011) [74]

^a^Different tools use either the term risk of bias or quality. We have reported the findings consistently with the terminology used in the individual tool

### Data analysis

Data were summarised in evidence tables and synthesised narratively for use of warning systems, compliance, effects of systems on patient outcomes, development and validation of systems, and cost-effectiveness studies. For the effects of systems on patient outcomes, a meta-analysis was planned but was not performed due to the limited number of studies (*n* = 1). For validation studies, we provided results for AUROC (area under the receiver operating characteristic curve) [17]. It equals one for a perfect test and 0.5 for a completely uninformative test. For health economics studies, we planned to examine the cost-effectiveness but no such studies were identified. The GRADE (Grades of Recommendation, Assessment, Development and Evaluation) approach was used to assess the certainty of the body of evidence for effects of systems on patient outcomes.

## Results

A total of 6397 records were identified. After removal of duplicates, 1147 database records were screened by title/abstract. Full texts of 83 records were assessed of which 43 studies (44 records) were included. The most common reason for exclusion was ‘non ED setting’ (*n* = 24). One study in Chinese was identified but the abstract was in English and presented relevant data that we included [18]. Five studies of the 56 screened additional resources were included. The results of the search/selection are presented in Fig. 1.

### Risk of bias and quality of reports

Three of the four descriptive studies assessing the extent of early warning system use in EDs were judged to be of fair quality [19–21] and one of poor quality [22]. The five descriptive studies assessing compliance with using early warning systems were assessed as being of good [23–25] and fair quality [26, 27]. The single effectiveness study was rated as having high ROB [28]. Eight studies that developed and validated a system (in the same sample) were rated as having low (*n* = 6) [29–34] and unclear (n = 2) [35, 36] ROB. The 28 studies that validated an existing system in a new cohort were judged as having an overall low (*n* = 16) [37–52], unclear (*n* = 9) [18, 53–60] and high ROB (*n* = 3) [61–63]. The domains of selection bias and factor measurement were most commonly rated as unclear ROB because they did not specify the methods of sampling (*n* = 10) [18, 36–38, 47, 48, 54, 58–60] or did not state cut-off values used (*n* = 12) [31, 33–35, 42, 46, 49, 57, 58, 60, 61, 63]. One study also did not pre-specify the outcomes clearly [59]. One scoping review of predictive ability of early warning systems was rated of good quality [64]. We have provided full details of the ROB and quality of reports in Additional file 3.

### Extent of use and compliance with early warning systems and track and trigger systems (1)

Four studies described the use of early warning systems within the ED and five studies examined compliance. The studies examining the extent of use collected data from medical records [19], a survey [20], a web-survey [21], and through participatory action research [22]. Considine et al. [19] described a pilot study of a 4-parameter system in the ED of a hospital in Australia and found that nurses made 93.1% of activations, the most common reasons being respiratory (25%) and cardiac (22.5%) and the median time between documenting physiological abnormalities and ED early warning system activation was 5 min (range 0–20). A survey in 2012 of 145 (57% response rate) clinical leads of EDs in the UK showed that 71% used an early warning system, most commonly the MEWS (80%) [20]. A survey in seven jurisdictions in Australia, found that 20 of 220 hospitals had a formal rapid response system in the ED but the prevalence of early warning systems in EDs was not reported [21]. Coughlan et al. [22] reported insufficient information in a conference abstract. The findings of these four studies demonstrate that multiple early warning systems are available and the extent of their use in the ED may vary geographically but limited data precludes comparisons between countries.

Three retrospective studies [23–25], one prospective study [27] and one audit (before and after early warning system implementation) [26] examined compliance with recording early warning system parameters. There was large variation in compliance ranging from 7% to 66% and factors such as patients’ triage category, age, gender, number of medications, length of hospital stay and the level of crowding in ED affected compliance with early warning systems [24]. Christensen et al. [23] reported a rate of 7% (22/300) of calculated scores in the clinical notes; however, 16% of records included all five vital signs. Heart rate (HR), shortness of breath (SOB) and loss of consciousness (LOC) were reported in 90–95% of records. Compliance with escalation of care varied; all nine patients that met the trauma call activation criteria had triggered a trauma call but only 24 of the 48 emergency call activation criteria had been responded to. Austen et al. [25] found a higher compliance with 66% of records containing an aggregate score, although only 72.6% were accurate. In an audit, the pre-implementation rate (30%) of abnormal vital sign identification was significantly lower than the post-implementation (53.5%) rate (*p* = 0.007) but no details of the implementation strategy were described [26]. Wilson et al. [27] compared the TTS scores recorded in charts with scores calculated retrospectively and found that 60.6% of charts contained at least one calculated TTS score but 20.6% (*n* = 211) were incorrect. This was mainly because of incorrect assignment of the score to an individual vital sign, which led to underscoring and reduced escalation activation. Hudson et al. [26] found that using a standardised emergency activation chart resulted in a higher percentage of abnormal vital signs recording (*p* = 0.007).

### Effects of early warning systems and track and trigger systems (2)

One non-randomised controlled design compared the effect of the MEWS (*n* = 269), recorded by emergency nurses every four hours, with clinical judgment (*n* = 275) in patients who are waiting for in-patient beds in the ED of a large hospital in Hong Kong [28]. It found that the MEWS might increase the rate of activating a critical pathway (1 per 10 patients with a MEWS >4 versus 1 in 20 patients based on clinical judgement) but might make little or no difference to the detection of deterioration or adverse events (0.4% is both groups). We assessed the overall body of evidence as very low quality (GRADE) due to serious imprecision and high ROB (Additional file 3).

### Development & Validation studies of early warning systems and track and trigger systems (3)

A scoping review by Challen et al. [64] identified 119 tools related to outcome prediction in ED; however, the majority were condition-specific tools (*n* = 94). They found the APACHE II score to have the highest reported AUROC curve (0.984) in patients with peritonitis.

Of the 36 primary development and/or validation studies, 13 were retrospective, 22 were prospective studies and one was a secondary analysis of a Randomised Controlled Trial (RCT) [48]. Eight studies developed and validated (in the same sample) an early warning system, while 28 validated an existing system in a different sample. Three studies included a random sample [30, 39, 43] and participants in the remaining studies were recruited consecutively or the sampling strategy was not stated clearly.

A total of 28 early warning systems were developed and/or validated. Churpek et al. [65] classified early warning systems into single-parameter systems, multiple-parameter systems and aggregate weighted scores. The early warning systems examined in the studies included primarily aggregate weighted scores (Table 3).

**Table 3:** Types of scores developed and/or validated in the included studies

Types of scores examined in the included development/validation studies	
Single-parameter systems	Aggregate weighted scores
ED Critical Instability Criteria (ED CIC) [39] Emergency severity index (ESI) [32]	Acute Physiology and Chronic Health Evaluation score (APACHE II) [31, 33, 52, 59] Assessment Score for Sick patient Identification and Step-up in Treatment (ASSIST) [50] Bispebjerg EWS (BEWS) [30] Charlson comorbidity index (CCI) [32, 38, 60] Early Warning Score (EWS) [55] Logistic Organ Dysfunction System (LODS) [48] Mainz Emergency Evaluation Score (MEES) [35] Modified Early Warning Score (MEWS) [18, 29, 31, 32, 35–38, 41–44, 50, 51, 54, 56–58, 60, 63] MEWS plus [43] Modified REMS (mREMS) [45] Morbidity Probability Model at admission (MPMO II) [48] National Early Warning Score (NEWS) [35, 40, 47, 49, 53] National Early Warning Score including Lactate (NEWS-L) [47] Patient Status Index (PSI) [61] Predisposition, Insult/Infection, Response, and Organ dysfunction model (PIRO) [59] Prince of Wales ED Score (PEDS) [31, 35] Rapid Acute Physiology Score (RAPS) [33, 34] Rapid Emergency Medicine Score (REMS) [29, 31, 33–35, 37] Revised Trauma Score (RTS) [31] Sequential Organ Failure Assessment (SOFA) [52] Simple Clinical Score (SCS) [35] New Simplified Acute Physiology Score (SAPS II) [48, 52] The Resuscitation Management score (THERM) [35] Triage Early Warning Score (TEWS) [62] VitalPAC Early Warning Score (VIEWS) [41] VitalPAC Early Warning Score-Lactate (VIEWS-L) [46]

No multiple parameter systems were identified

The most common outcomes examined were in-hospital mortality (*n* = 21), admission to ICU (*n* = 12), mortality (not specified where or during a specific follow up time frame possibly beyond hospital discharge) (*n* = 11), hospital admission (*n* = 7), and length of hospital stay (*n* = 5). Only one study measured the number of patients identified as critically ill as outcome [50]. Overall, the APACHE II score, PEDS, VIEWS-L, and THERM scores appeared relatively better at predicting mortality and ICU admission. The MEWS was the most commonly assessed tool and the cut-off value used was 4 or 5, with the exception of Dundar et al. [41] who found an optimal cut-off of 3 for predicting hospitalisation. To synthesise the findings, studies were categorised into three groups according to the degree of differentiation of the ED patient group: a patient group in a specific triage category(ies), a patient group with a certain (suspected) condition or an undifferentiated patient group. Findings are presented in Tables 4, 5 and 6 and full details are provided in Additional file 4.

**Table 4:** Evidence table: Development and validation studies – Patient groups differentiated by triage category

Authors (year), country, ROB	No of participants	Tool (cut-off if provided)	Results by outcome
Alam et al. (2015) [53], the Netherlands Risk of bias: Unclear	274 at time zero (T0); 247 1 h later (T1); 133 at discharge from the ED (T2).	NEWS	Hospital admission (*n* = 130) T0: AUROC 0.66 (95% CI 0.60–0.73) T1: AUROC 0.69 (95% CI 0.62–0.75) T2: AUROC 0.70 (95% CI 0.61–0.79) Length of stay NEWS associated with length of stay at all 3 time points (*p* < 0.001). (AUROC not provided) ICU admission (*n* = 10) NEWS associated with ICU admission at all 3 time points (T0: *p* = 0.003; T1: *p* = 0.001; T2: *p* = 0.046). (AUROC not provided) 30-day Mortality (*n* = 11) T0: AUROC 0.77 (95% CI 0.62–0.92) T1: AUROC 0.87 (95% CI 0.77–0.96) T2: AUROC 0.77 (95% CI 0.57–0.97).
Armagan et al. (2008) [54], Turkey Risk of bias: Unclear	309	MEWS	MEWS (cut-off >4) Admission to hospital: adjusted OR 1.56 (95% CI 0.93–2.98) Admission to ICU: adjusted OR 1.95 (95% CI 1.04–366.00) Death in ED: adjusted OR 35.13 (95% CI 4.58–269.40) Death in hospital: adjusted OR 14.80 (95% CI 5.52–39.70)
Bulut et al. (2014) [37], Turkey Risk of bias: Low	2000	REMS MEWS	In-hospital mortality MEWS AUROC: 0.63 (95% CI 0.61–0.65) REMS AUROC: 0.71 (95% CI 0.67–0.72) Performance of REMS was higher (*p* < 0.001) Discharge vs hospitalisation MEWS: AUROC 0.57 (95% CI 0.55–0.59) REMS: AUROC 0.64 (95% CI 0.62–0.66) Performance of REMS was higher (*p* < 0.001) Admission to ICU/HDU MEWS: AUROC 0.54 (95% CI 0.52–0.56) REMS: AUROC 0.59 (95% CI 0.57 to 0.61) Performance of REMS was higher (*p* < 0.001)
Cattermole et al. (2009) [31], Hong Kong Risk of bias: Low	330	PEDS RTS REMS MEWS APACHE II	Death or admission to ICU within 7 days of ED attendance PEDS: AUROC 0.90 (95% CI 0.87–0.94) APACHE II: AUROC 0.73 (95% CI 0.68–0.78) RTS: AUROC 0.75 (95% CI 0.70–0.79) REMS: AUROC 0.70 (95% CI 0.64–0.75) MEWS: AUROC 0.76 (95% CI 0.71–0.81) 30-day mortality PEDS: AUROC 0.90 (95% CI 0.86–0.93) APACHE II: AUROC 0.84 (95% CI 0.79–0.88) RTS: AUROC 0.77 (95% CI 0.72–0.81) REMS: AUROC 0.77 (95% CI 0.72–0.82) MEWS: AUROC 0.75 (95% CI 0.70–0.80)
Cattermole et al. (2013) [35], Hong Kong Risk of bias: Unclear	234	THERM PEDS MEWS SCS REMS MEES NEWS	Admitted to ICU or death within 7 days PEDS: AUROC 0.75 (95% CI 0.69 to 0.80) MEES: AUROC 0.75 (95% CI 0.69 to 0.80) MEWS: AUROC 0.73 (95% CI 0.67 to 0.79) NEWS: AUROC 0.71 (95% CI 0.64 to 0.76) REMS: AUROC 0.70 (95% CI 0.64 to 0.76) SCS: AUROC 0.70 (95% CI 0.64 to 0.76) THERM: AUROC: 0.84 (95% CI 0.79 to 0.88)
Christensen et al. (2011) [30], Denmark Risk of bias: Low	162	BEWS (≥ 5)	Death within 48 h of arrival Sensitivity 83.0%, Specificity 83.0% ICU admission within 48 h of arrival Sensitivity 50.0%, Specificity 81.0% Critically ill Sensitivity 63.0%, Specificity 82.0%
Gu et al. (2015) [18], China Risk of bias: Unclear (Data from abstract in English)	176	MEWS (≥ 5)	3-days mortality (*n* = 41) OR = 1.7 (95% CI 0.6–4.5), *P* = 0.3 All death (*n* = 58) OR 5.5 (95% CI 2.8–10.9), *P* < 0.001 ICU transfer, cardio-pulmonary resuscitation and death (*n* = 74) OR 5.4 (95% CI 2.8–10.4), *P* < 0.001
Ho et al. (2013) [44], Singapore Risk of bias: Low	1024	MEWS (≥4)	Mortality AUROC: 0.68 Admission AUROC: 0.5
Hock Ong et al. (2012) [57], Singapore Risk of bias: Unclear	925	MEWS	Cardiac arrest AUROC: 0.7 Death after admission AUROC: 0.7
Keep et al. (2015) [49], UK Risk of bias: Low	500	NEWS (≥3)	Prediction of Septic Shock AUROC 0.90 (95% CI 0.84–0.94).
Lui et al. (2014) [36], Singapore Risk of bias: Unclear	564	MEWS (≥1)	Mortality, cardiac arrest, sustained ventricular tachycardia, and hypotension requiring inotropes or intraaortic balloon pump insertion within 72 h of arrival at the ED AUROC: 0.67 (0.54–0.81)
Wilson et al. (2016) [61], UK Risk of bias: High	472 adults	PSI	PSI true alerts Detected by paper TTS: 4 Detected by electronic TTS: 17 Detected by PSI: 15 Detected by eTTS, not PSI: 5 Detected by PSI, not eTTS: 3 PSI false alerts False alert rate: 1.13 alerts/bed-day (49 false alerts from 39 patients).

**Table 5:** Evidence table: Development and validation studies – Patient groups differentiated by (suspected) condition

Authors (year), country	Participants	Tool (cut-off if provided)	Results
Albright et al. (2014) [29], USA Risk of bias: Low	850 pregnant & post partum women with suspected SIRS/sepsis	MEWS (≥5) REMS (≥6)	ICU Admission within 48 h prediction MEWS: Sensitivity 100.0%, Specificity 77.6% REMS: Sensitivity 77.8%, Specificity 93.3%
Cildir et al. (2013) [38], Turkey Risk of bias: Low	230 diagnosed with community acquired sepsis.	CCI (>5) MEWS (≤5)	28-day mortality CCI: AUROC 0.65 (p = 0.001) MEWS: AUROC 0.61 (*p* = 0.008) 28-day mortality (*n* = 64 with sepsis) CCI: AUROC 0.65 (*p* = 0.18) MEWS:AUROC 0.57 (*p* = 0.48) 28-day mortality (*n* = 166 with severe sepsis) CCI: AUROC 0.62 (*p* = 0.006) MEWS: AUROC 0.60 (p = 0.04)
Considine et al. (2015) [39], Australia Risk of bias: Low	600 adult with presenting with SOB, chest pain or abdominal pain	ED CIC	Episodes of unreported clinical deterioration T0 (Clinical decision making) (86.7%); T1 (Escalation of care protocol) (68.8%); T2 (Escalation of care protocol, single parameter TTS chart) (55.3%); T3 (Escalation of care protocol, single parameter TTS chart (year 2012)) (54.0%); (*p* = 0.14).
Corfield et al. (2014) [75] (and related conference abstract Corfield et al. (2012) [40], Scotland Risk of bias: Low	2003 with sepsis (suspected or confirmed within 2 days of attendance and 2 or more of sepsis criteria)	NEWS (≥9 versus 0–4)	ICU (within 2 days) OR 5.76 (95% CI 3.22–10.31; p = 0.00) Mortality (30 days) OR 5.64 (95% CI 3.70–8.60; p = 0.00) Combined (ICU and/or mortality) 9–20: OR 5.78 (95% CI 4.02–8.31; p = 0.00) Cut-off point with highest Youden’s Index: NEWS 9
Geier et al. (2013) [32] Germany Risk of bias: Low	151 with suspected sepsis	ESI MEWS CCI Score	In-hospital mortality ESI: Sensitivity 0.73, Specificity 0.0 MEWS: Sensitivity 0.43, Specificity 0.74 CCI: Sensitivity 0.82, Specificity 0.64
Howell et al. (2007) [45], USA Risk of bias: Low	2132 with suspected infection	mREMS	28-day in-hospital survival AUROC 0.80 (95% CI 0.75–0.85)
Jo et al. (2013) [46], Korea Risk of bias: Low	299 patients with blunt trauma, Injury severity score ≥ 9	VIEWS-L	In-hospital mortality AUROC: 0.83 (95% CI 0.77–0.91)
Jo et al. (2016) [47], Korea Risk of bias: Low	553 with pneumonia	NEWS-L score (≥3.1) NEWS (≥5)	In-hospital mortality NEWS-L: AUROC 0.73 (0.66–0.80) NEWS: AUROC 0.70 (0.63–0.77)
Jones et al. (2005) [48], USA Risk of bias: Low	91 with initial ED vital signs consistent with shock	SAPS II MPM0 II LODS	In-hospital mortality SAPS II: AUROC 0.72 (95% CI 0.57–0.87) MPM0 II: AUROC 0.69 (95% CI 0.54–0.84) LODS: AUROC 0.60 (95% CI 0.45–0.76)
Nguyen et al. (2012) [59], USA Risk of bias: Unclear	541 with severe sepsis	PIRO APACHE II	In-hospital mortality PIRO: AUROC 0.71 (95% CI 0.66–0.75) APACHE II: AUROC 0.71 (95% CI 0.66–0.76)
Vorwerk et al. (2009) [51], UK Risk of bias: Low	307 with sepsis	MEWS (≥5) Blood lactate (≥4 mmol/l)	28-day mortality MEWS: AUROC 0.72 (95% CI 0.67 to 0.77) Lactate: AUROC 0.62 (0.54 to 0.70)
Williams et al. (2016) [52], Australia Risk of bias: Low	8871 with presumed infection	SAPS II) SOFA APACHE II	30-day mortality APACHE II: AUROC 0.90 (0.88–0.91) SAPS II: AUROC 0.90 (0.89–0.92) SOFA: AUROC 0.86 (0.84–0.88)

**Table 6:** Evidence table: Development and validation studies – Undifferentiated patient groups

Authors (year), country	Participants	Tool (cut-off if provided)	Results
Burch et al. (2008) [63], South Africa Risk of bias: High	790	MEWS	Hospital admission MEWS 0–2 (ref) MEWS 3–4: RR 1.3 (95% CI 1.1 to 1.6) MEWS ≥5: RR 1.7 (95% CI 1.5 to 2.0) In-hospital mortality MEWS 0–2 (ref) MEWS 3–4: RR 2.8 (95% CI 1.7 to 4.8) MEWS ≥5: RR 4.6 (95% CI 2.7 to 7.8)
Correia et al. (2014) [55], Portugal Risk of bias: Unclear	65	EWS	Length of hospital stay & Mortality Score at 24 h and 12 h seemed to predict both length of stay and mortality (*p* < 0.05). The EWS would have increased early medical attention by 40% if a threshold of ≥3 was used.
Dundar et al. (2015) [41], Turkey Risk of bias: Low	671	MEWS VIEWS	Hospitalisation MEWS (≥3): AUROC 0.73 (95% CI 0.69–0.77) VIEWS (≥6): AUROC 0.76 (95% CI 0.72–0.79) In-hospital mortality MEWS (≥4): AUROC 0.89 (95% CI 0.84–0.94) VIEWS (≥8): AUROC 0.90 (95% CI 0.86–0.94)
Eick et al. (2015) [42], Germany Risk of bias: Low	5730	MEWS	In-hospital mortality AUROC: 0.71 (0.67–0.75; *p* < 0.001)
Graham et al. (2007) [56], Hong Kong Risk of bias: Unclear (Conference abstract)	413	MEWS (>4)	In-hospital mortality OR 8.3 (95% CI 1.1–60.4), *p* = 0.013 ED re-attendance within 48 h OR 45.2 (95% CI 3.4–568.9), *p* < 0.0001
Heitz et al. (2010) [43], USA Risk of bias: Low	280	MEWS Max (≥4) MEWS plus	Need for higher level of care or mortality within 24 h MEWS Max: AUROC 0.73 (95% CI, 0.66–0.79) MEWS Plus: AUROC 0.76 (95% CI, 0.69–0.82)
Junhasavasdiku et al. (2012) [58], Thailand Risk of bias: Unclear	381	MEWS	Mortality MEWS at ED was associated with mortality (*p* < 0.001)
Naidoo et al. (2014) [62], South Africa Risk of bias: High	265	TEWS	Discharge within 24 h of admission, admission to a ward, admission to an intensive care unit (ICU), and death in hospital. TEWS <7: 53.7% discharged; no admitted to ICU; none died. TEWS ≥7: 18.7% discharged; 3 admitted to ICU; 4 died
Olsson et al. (2003) [33], Sweden Risk of bias: Low	1027	APACHE II RAPS REMS	Mortality REMS: AUROC: 0.91 ± 0.02 RAPS: AUROC: 0.87 ± 0.02 APACHE II: AUROC: 0.90 ± 0.02
Olsson et al. (2004) [34], Sweden Risk of bias: Low	11,751	RAPS REMS	Mortality RAPS: AUROC: 0.65 ± 0.02 REMS: AUROC: 0.85 ± 0.01
Subbe et al. (2006) [50], UK Risk of bias: Low	(a) 53 unselected; (b): 49 ICU admission; (c): 49 ED admission, transferred to ward then ICU	MEWS (>2) ASSIST (>3) MET (=1) MTS (orange or red)	Patients identified as critically ill (at risk of deterioration) MTS: Sensitivity: (a) 15%; (b) 96%; (c) 65% MEWS: Sensitivity (a): 8%; (b) 77%; (c) 55% ASSIST: Sensitivity (a): 0%; (b) 22%; (c) 16% MET: Sensitivity (a) 0%; (b) 2%; (c) 7%
Wang et al. (2016) [60], Taiwan Risk of bias: Unclear	99	CCI MEWS	Survival to discharge CCI: Adjusted OR 0.57 (95% CI 0.38–0.84); *p* = 0.005 Peri-arrest MEWS: Adjusted OR 0.77 (95% CI 0.60–0.97); *p* = 0.028

Twelve of the 36 validation studies only included participants in (a) specific triage category(ies) (Table 4). Triage systems varied but included categories of patients that were critically ill (e.g. Manchester triage system I-III, Patient acuity category scale 1 or 2) or were admitted to the resuscitation room. In predicting mortality, the AUROC for the MEWS ranged from 0.63 to 0.75 [36, 37, 44, 57], from 0.70–0.77 for REMS [31, 37], 0.77–0.87 for NEWS [53], 0.90 for PEDS, 0.83 for APACHE II, and 0.77 for RTS [31]. Predicting ICU admission, the AUROC were 0.54 [37] and 0.49 [44] for MEWS and 0.59 for REMS [37], while to predict hospital admission the AUROC for NEWS was 0.66–0.70 [53]. Cattermole et al. [31] and Cattermole et al. [35] used a combined outcome of death and ICU admission and found an AUROC of 0.76 and 0.73 for MEWS, 0.90 and 0.75 for PEDS, 0.73 for APACHE II, 0.75 for RTS, 0.70 and 0.70 for REMS, 0.75 for MEES, 0.71 for NEWS, 0.70 for SCS and 0.84 for THERM. One study assessed the prediction of septic shock by NEWS (AUROC 0.89) [49].

Eleven other studies (12 records; Table 5) included a differentiated patient group with a specific (suspected) condition. Five studies only included patients with (suspected) sepsis [29, 32, 38, 40, 51, 59]. Other study populations were restricted to patients with trauma [46], suspected infection [45, 52], pneumonia [47] or who had signs of shock [48]. Assessing the predictive ability of systems to predict mortality, MEWS had an AUROC of 0.61 [38] and 0.72 [51], CCI of 0.65 [38], mREMS of 0.80 [45], NEWS of 0.70 [47], NEWS-L of 0.73 [47], VIEWS-L of 0.83 [46], SAPS II of 0.72 [48] and 0.90 [52], MPMO II of 0.69 [48], LODS of 0.60 [48], PIRO of 0.71 [59], APACHE II of 0.71 [59] and 0.90 [52], and SOFA of 0.86 [52].

The remaining 13 studies assessed early warning systems in an undifferentiated ED population (Table 6). The AUROC to predict mortality was 0.71 [42], 0.73 [43], and 0.89 [41] for MEWS, 0.76 for MEWS plus [43], 0.91 [33] and 0.85 [34] for REMS, 0.87 [33] and 0.65 [34] for RAPS and 0.90 for APACHE II [33].

We did not identify studies that examined the cost effectiveness of early warning systems or TTS in EDs, nor did we find any studies evaluating related educational programmes (objectives (4) and (5)).

## Discussion

Multiple early warning systems were identified but the extent to which they are used in the ED seems to vary across countries for which data were available in the nine included descriptive studies. Moreover, incorrect score calculation was common. Compliance with recording aggregate scores was relatively low although the vital signs HR and BP were usually recorded. This finding emphasises the importance of effective implementation strategies. However, we did not identify any studies examining educational programmes for early warning systems. Existing guidelines regarding the use of early warning systems to monitor acute patients in hospital do include educational tools but are not specific to the ED [7, 8]. Using early warning systems in the ED would likely require contextual adaptation to the ED environment, for example broadening of the ranges of physiological parameters to reflect acutely unwell patients’ physiology. In implementing an early warning system in the ED, staff training could consist of a joined core package applicable to any service supplemented by an ED specific component. The performance of early warning systems in the ED will also depend on the time patients spend in the ED, which varies substantially between countries.

Evidence from 36 validation and development studies demonstrated that early warning systems used in ED settings seem to be able to predict adverse outcomes, based on the AUROC, but there is variability between studies. All but two early warning systems were aggregated scores, which limited the ability to compare between single, multiple parameter and aggregated scores. The APACHE II score, PEDS, VIEWS-L, and THERM scores were relatively best at predicting mortality and ICU admission, providing excellent discrimination ability (AUROC >0.8) [66]. The MEWS was the most commonly assessed system but findings suggest a relatively lower ability to predict mortality and ICU admissions compared to the four scores mentioned above, with only some studies indicating acceptable discriminatory ability (AUROC >0.7) and other studies indicating a lack of discriminatory ability (AUROC <0.7) [66], especially for the outcome of ICU admission. The exception was one low ROB study that found excellent discriminatory ability of MEWS for the outcome in-hospital mortality (AUROC 0.89) [41]. This was the only study that examined the MEWS in an undifferentiated sample, which could contribute to this observed difference. However, the ability of early warning systems to predict adverse outcomes does not mean that they are effective at preventing adverse outcomes through early detection of deterioration. Only one study addressed this question and it found that the introduction of an early warning system may have little or no difference in detecting deterioration or adverse events; however, the evidence was of very low quality making it impossible to draw any strong conclusions. The effectiveness of early warning systems also highly depends on an appropriate response to such systems. If effective, the role of early warning systems in the ED could primarily be to assist with patient and resource management in the post-triage phase, when the time for patients to see a treating clinicians is prolonged (overcrowding). They could also provide additional information to help determine who to refer to critical care admission or to guide discharge from the ED, but this is currently not generally their purpose in places where they have been implemented in the ED. Recent studies also show that additional laboratory data (e.g. D-dimer, lactate) might enhance the performance of early warning systems in predicting adverse outcome [67, 68].

The cost effectiveness of early warning systems remains unclear. While it is clear that implementing early warning systems requires a healthcare resource investment, the degree to which such systems may or may not result in cost savings remains unclear, particularly since the effectiveness of early warning systems in the ED is uncertain. The limited evidence base suggests that early warning systems might be effective in, for example, identifying deteriorating patients. This could result in improved patient outcomes and, should these effects exist, the potential healthcare cost savings could go towards funding, at least to some degree, their implementation. While this theory is open to question, it highlights the need to conduct primary research studies that directly evaluate their cost effectiveness. Such studies should focus on the monitoring of resource use, costs and patient outcomes in order to determine whether early warning systems are likely to deliver good value for money.

### Limitations

We did not translate reports although only one non-English study was identified. We could not pool findings of the validation studies due to clinical heterogeneity; however, the AUROC were provided to inform accuracy of the models. Strengths of the review lie in its thorough search strategy, its scope and inclusion of different designs to best address the objectives and in its rigorous methodology with dual independent screening and quality assessment.

## Conclusions

There are a lack of high quality RCTs examining the effects of using early warning systems in the ED on patient outcomes. The cost-effectiveness of such interventions, compliance, the effectiveness of related educational programmes and barriers and facilitators to implementation also need to be examined and reported as presently there is a clear lack of such evidence.

## Additional files

Additional file 1: Search strategies. This additional file contains a detailed description of the search strategies for the individual databases and other resources searched. (DOCX 49 kb)

Additional file 2: Data Extraction. The elements that were extracted for each study type included in this review. (DOCX 33 kb)

Additional file 3: Risk of bias and methodological quality assessment. A detailed description of the risk of bias/quality assessment of the included studies. (DOCX 53 kb)

Additional file 4: Development and validation studies, additional information. In depth study information on the included studies developing and/or validating early warning system(s). (DOCX 69 kb)

## Abbreviations

AGREE II: Appraisal of Guidelines for Research & Evaluation
AMSTAR: Assessing the Methodological quality of Systematic Reviews
APACHE II: Acute Physiology and Chronic Health Evaluation score
ASSIST: Assessment Score for Sick patient Identification and Step-up in Treatment
AUROC: Area under the Receiver Operating Curve
BEWS: Bispebjerg Early Warning Score
BP: Blood Pressure
CCI: Charlson comorbidity index
CI: Confidence Interval
CINAHL: Cumulative Index to Nursing and Allied Health Literature
CURB-65: Confusion, Urea, Respiratory rate, Blood pressure, Age 65 or older
DIST: An Euclidean Distance-based Scoring System
ED CIC: ED Critical Instability Criteria
ED: Emergency Department
EDWIN: Emergency Department Work INdex
EPOC: Effective Practice and Organisation of Care
ESI: Emergency severity index
ESS: Proposed Ensemble-Based Scoring System
eTTS: Electronically calculated Track & Trigger Score
EWS: Early Warning Score
GRADE: Grading of Recommendations Assessment, Development and Evaluation
HDU: High Dependency Unit
HR: Heart Rate
HRV: Heart Rate Variability
ICER: Incremental Cost-Effectiveness Ratios (ICERs)
ICU: Intensive Care Unit
IMEWS*: Irish Maternity Early Warning System
LOC: Loss of Consciousness
LODS: Logistic Organ Dysfunction System
MEDS: Mortality in emergency department sepsis
MEES: Mainz Emergency Evaluation Score
MeSH: Medical Subject Headings
MEWS plus: Modified Early Warming Score plus
MEWS*: Modified Early Warning Score
MPM0 II: Morbidity Probability Model at admission
mREMS: Modified Rapid Emergency Medicine Score
MTS: Manchester Triage System
NEDS: Nationwide Emergency Department Sample
NEWS (Ireland): Irish National Early Warning Score
NEWS: National Early Warning Score
NEWS-L: National Early Warning Score + Lactate
NICE: National Institute for Health and Care Excellence
OR: Odds Ratio
PACS: Patient acuity category scale
PARS: Patient at Risk Score
PEDS: Prince of Wales ED Score
PEWS: Paediatric Early Warning System
PIRO: Predisposition, Insult/Infection, Response, and Organ dysfunction
PSI: Patient Status Index
QALYs: Quality Adjusted Life Years
RAPS: Rapid Acute Physiology Score
REMS: Rapid Emergency Medicine Score
ROB: Risk of Bias
ROC: Receiver Operating Curve
RR: Risk Ratio
RTS: Revised Trauma Score
SAPS II: New Simplified Acute Physiology Score
SCS: Simple Clinical Score
SD: Standard Deviation
SIRS: Systemic Inflammatory Response Syndrome
SOFA: Sequential Organ Failure Assessment
SOS: Sepsis in Obstetrics Score
SSSS: Severe Sepsis and Septic Shock score
TEWS: Triage Early Warning Score
THERM: The Resuscitation Management score
TRISS: Trauma – Injury Severity Score
TTS: Track and Trigger System
VIEWS: VitalPAC Early Warning Score
VIEWS-L: VitalPAC Early Warning Score-Lactate
