# Supplementary material for: The effectiveness of physiologically based early warning or track and trigger systems after triage in adult patients presenting to emergency departments: a systematic review
Source: BMC Emerg Med. 2017 Dec 6;17:38. doi: 10.1186/s12873-017-0148-z (PMC5719672; doi:10.1186/s12873-017-0148-z)
Supplement: Supplementary file 1 — Search strategies. This additional file contains a detailed description of the search strategies for the individual databases and other resources searched. (DOCX 49 kb) [file 12873_2017_148_MOESM1_ESM.docx]

**Additional File 1: Search strategies**

| **Cochrane Library Search Strategy** | | |
| --- | --- | --- |
| **Platform: John Wiley and Sons** | | |
| **Date of search: 04/03/2016** | | |
| **ID** | **Search** | **Hits** |
| #1 | MeSH descriptor: [Emergency Service, Hospital] explode all trees | 2078 |
| #2 | Emergency near/1 (care or ward or wards or department or departments or unit or units or room or room or medic* or health or healthcare or hospital or service or services or centre or centres or center or centers or treatment or treatments or outpatient or outpatients) | 12379 |
| #3 | Casualty near/2 (care or ward or wards or department or departments or unit or units or room or room or service or services or centre or centres or center or centers or outpatient or outpatients) | 68 |
| #4 | Trauma near/2 (care or ward or wards or department or departments or unit or units or room or room or medic* or hospital or service or services or centre or centres or center or centers or outpatient or outpatients) | 1505 |
| #5 | Triage near/2 (care or ward or wards or department or departments or unit or units or room or room or medic* or hospital or service or services or centre or centres or center or centers or outpatient or outpatients) | 98 |
| #6 | "accident and emergency" or "accident & emergency" or A&E or "A & E" or "A and E" | 1431 |
| #7 | {or #1-#6} | 14493 |
| #8 | Warning near/2 (early or system or systems or score or scores) | 168 |
| #9 | Trigger near/2 track | 6 |
| #10 | Trigger near/4 (score or scores or scoring) | 20 |
| #11 | Escalation near/2 (protocol or protocols or policy or policies or procedure or procedures or guideline or guidelines or guidance) | 25 |
| #12 | EWS or MEWS | 75 |
| #13 | POTTS | 177 |
| #14 | {or #8-#13} | 443 |
| #15 | adult or adults or adulthood | 402614 |
| #16 | #7 and #14 and #15 | 38 |

| **Medline Search Strategy** | | |
| --- | --- | --- |
| **Platform: Ovid MEDLINE In-Process & Other Non-Indexed Citations and Ovid MEDLINE 1946 to Present** | | |
| **Date of Search: 04/03/2016** | | |
| **ID** | **Search** | **Hits** |
| #1 | emergency service, hospital.sh. | 48807 |
| #2 | (Emergency adj (care or ward or wards or department or departments or unit or units or room or room or medic* or health or healthcare or hospital or service or services or centre or centres or center or centers or treatment or treatments or outpatient or outpatients)).af. | 197858 |
| #3 | (Casualty adj2 (care or ward or wards or department or departments or unit or units or room or room or service or services or centre or centres or center or centers or outpatient or outpatients)).af. | 1309 |
| #4 | (Trauma adj2 (care or ward or wards or department or departments or unit or units or room or room or medic* or hospital or service or services or centre or centres or center or centers or outpatient or outpatients)).af. | 47447 |
| #5 | (Triage adj2 (care or ward or wards or department or departments or unit or units or room or room or medic* or hospital or service or services or centre or centres or center or centers or outpatient or outpatients)).af. | 1679 |
| #6 | ("accident and emergency" or "accident & emergency" or A&E or "A & E" or "A and E").af. | 1886693 |
| #7 | 1 or 2 or 3 or 4 or 5 or 6 | 2093553 |
| #8 | (Warning adj2 (early or system or systems or score or scores)).af. | 4296 |
| #9 | (Trigger adj2 track).af. | 67 |
| #10 | (Trigger adj4 (score or scores or scoring)).af. | 56 |
| #11 | (Escalation adj2 (protocol or protocols or policy or policies or procedure or procedures or guideline or guidelines or guidance)).af. | 168 |
| #12 | (EWS or MEWS).af. | 2180 |
| #13 | POTTS.af. | 5046 |
| #14 | 8 or 9 or 10 or 11 or 12 or 13 | 11541 |
| #15 | (adult or adults or adulthood).af. | 4789831 |
| #16 | 7 and 14 and 15 | 362 |

| **Embase Search Strategy** | | |
| --- | --- | --- |
| **Platform: Elsevier** | | |
| **Date of Search: 22/02/2016** | | |
| **ID** | **Search** | **Hits** |
| #1 | 'emergency ward'/exp | 82019 |
| #2 | emergency NEAR/1 (care OR ward OR wards OR department OR departments OR unit OR units OR room OR rooms OR medic* OR health OR healthcare OR hospital OR service OR services OR center OR centers OR centre OR centres OR treatment OR treatments OR outpatient OR outpatients) | 317266 |
| #3 | casualty NEAR/2 (care OR ward OR wards OR department OR departments OR unit OR units OR room OR room OR service OR services OR centre OR centres OR center OR centers OR outpatient OR outpatients) | 1741 |
| #4 | trauma NEAR/2 (care OR ward OR wards OR department OR departments OR unit OR units OR room OR room OR medic* OR hospital OR service OR services OR centre OR centres OR center OR centers OR outpatient OR outpatients) | 51937 |
| #5 | triage NEAR/2 (care OR ward OR wards OR department OR departments OR unit OR units OR room OR room OR medic* OR hospital OR service OR services OR centre OR centres OR center OR centers OR outpatient OR outpatients) | 1827 |
| #6 | accident and emergency' OR 'accident & emergency' OR a&e OR 'a & e' OR 'a and e' | 168515 |
| #7 | #1 OR #2 OR #3 OR #4 OR #5 OR #6 | 512537 |
| #8 | warning NEAR/2 (early OR system OR systems OR score OR scores) | 5852 |
| #9 | trigger NEAR/2 track | 110 |
| #10 | trigger NEAR/4 (score OR scores OR scoring) | 116 |
| #11 | escalation NEAR/2 (protocol OR protocols OR policy OR policies OR procedure OR procedures OR guideline OR guidelines OR guidance) | 341 |
| #12 | ews OR mews | 4360 |
| #13 | potts | 4486 |
| #14 | #8 OR #9 OR #10 OR #11 OR #12 OR #13 | 14723 |
| #15 | adult OR adults OR 'adulthood' | 5624653 |
| #16 | #7 AND #14 AND #15 | 254 |

| **CINAHL Complete Search Strategy** | | |
| --- | --- | --- |
| **Platform: EBSCOhost** | | |
| **Date of Search: 04/03/2016** | | |
| **ID** | **Search** | **Hits** |
| S1 | (MH "Emergency Service") | 34352 |
| S2 | Emergency N1 (care OR ward or wards OR department or departments OR unit or units OR room or rooms OR medic* OR health OR healthcare OR hospital OR service or services OR center or centers OR centre or centres OR treatment or treatments OR outpatient or outpatients) | 99485 |
| S3 | Casualty N2 (care OR ward or wards OR department or departments OR unit or units OR room or rooms OR service or services OR center or centers OR centre or centres OR outpatient or outpatients) | 402 |
| S4 | Trauma N2 (care or ward or wards or department or departments or unit or units or room or room or medic* or hospital or service or services or centre or centres or center or centers or outpatient or outpatients) | 9842 |
| S5 | Triage N2 (care or ward or wards or department or departments or unit or units or room or room or medic* or hospital or service or services or centre or centres or center or centers or outpatient or outpatients) | 978 |
| S6 | "accident and emergency" or "accident & emergency" or A&E or "A & E" or "A and E" | 52174 |
| S7 | S1 OR S2 OR S3 OR S5 OR S6 | 149039 |
| S8 | Warning N2 (early or system or systems or score or scores) | 1131 |
| S9 | Trigger N2 track | 44 |
| S10 | Trigger N4 (score or scores or scoring) | 26006 |
| S11 | Escalation N2 (protocol or protocols or policy or policies or procedure or procedures or guideline or guidelines or guidance) | 37 |
| S12 | ews OR mews | 196 |
| S13 | potts | 184 |
| S14 | S8 OR S9 OR S10 OR S11 OR S12 OR S13 | 27422 |
| S15 | adult OR adults OR adulthood | 892275 |
| S16 | S7 AND S14 AND S15 | 653 |

| **Cost Effectiveness Resources** | | | |
| --- | --- | --- | --- |
| **Date of Search: 11/03/2016 (except * searched 04/03/2016)** | | | |
| **Website/Database** | **URL** | **Search Terms** | **Hits** |
| **Health Technology Assessment Database, NHS Economic Evaluation Database (NHSEED) & Health Economic Evaluation Database (HEED) via The Cochrane Library*** | [www.cochranelibrary.com](http://www.cochranelibrary.com/) | See Cochrane Library tab | n/a |
| **NHS Service Delivery and Organisation (SDO) Research and Development Programme** | [www.nets.nihr.ac.uk/programmes/hsdr](http://www.nets.nihr.ac.uk/programmes/hsdr) | patient deterioration emergency department | 0 |
|  |  | patient deterioration emergency | 4. After sifting = 0 |
|  |  | patient deterioration | 46. After sifting = 0 |
|  |  | early warning | 13. After sifting = 1 |
|  |  | track and trigger | 5. After sifting = 0 |
| **National Coordinating Centre for Health Technology Assessment (NCCHTA)** | [www.nets.nihr.ac.uk/programmes/hta](http://www.nets.nihr.ac.uk/programmes/hta) | patient deterioration emergency department | 0 |
|  |  | patient deterioration emergency | 4. After sifting = 0 |
|  |  | patient deterioration | 46. After sifting = 0 |
|  |  | early warning | 13. After sifting = 1. Same as result for website above, hence discarded |
|  |  | track and trigger | 5. After sifting = 0 |
| **NIHR-HTA Database** | <http://www.crd.york.ac.uk/CRDWeb/> | patient deterioration. Filters: HTA published and HTA in progress | 2 |

| **Guidance Resources** | | | |
| --- | --- | --- | --- |
| **Date of Search: 13/03/2016 (except * searched 11/03/2016)** | | | |
| **Website/ Database** | **URL** | **Search Terms** | **Hits** |
| **Department of Health (including National Clinical Guidelines)*** | via Google Advanced Search https://www.google.com/advanced_search | emergency adult* warning OR OR OR triage OR OR OR care OR OR OR trama OR OR OR trigger OR OR OR esclat* OR OR OR EWS OR OR OR MEWS OR OR OR POTTS . Site filter:health.gov.ie Region filter: Ireland | 495. After sifting first 200 hits = 4 . Plus 2 added from brief manual search. |
| **Health Service Executive (HSE)*** | via Google Advanced Search https://www.google.com/advanced_search | emergency adult* warning OR OR OR triage OR OR OR care OR OR OR trama OR OR OR trigger OR OR OR esclat* OR OR OR EWS OR OR OR MEWS OR OR OR POTTS. Site filter:hse.ie Region filter: Ireland | 1880. After sifting first 200 hits = 4. |
| **Health Information and Quality Authority (HIQA)** | [www.hiqa.ie](http://www.hiqa.ie/) | "emergency department patient deterioration" in keyword box | 92. After sifting = 0 |
| **National Institute for Health and Care Excellence (NICE)** | <https://www.nice.org.uk/guidance> | patient deterioration emergency department | 55. After sifting = 4 |
|  |  | "early warning" or "track and trigger" or ews or mews or potts | 38. After sifting = 5 |
| **NHS Evidence ( incorporating Scottish Intercollegiate Guidelines Network (SIGN) & Guidelines International Network (GIN))** | [https://www.evidence.nhs.uk](https://www.evidence.nhs.uk/) | "patient deterioration" and "emergency department" and ("early warning" or "track and trigger" or ews or mews or potts) | 17. After sifting = 1 |
|  |  | emergency department and ("early warning" or "track and trigger" or ews or mews or potts) | 419. After sifting = 3 |
| **Agency for Healthcare Research and Quality (AHRQ) National Guideline Clearinghouse** | [http://www.guideline.gov](http://www.guideline.gov/) | (triage or casualty or trauma or "emergency department")' and '("early warning" or trigger or ews or mews or potts)'  Filters: Adult (19 to 44 years) Aged (65 to 79 years) Aged, 80 and over | 76. after sifting = 0 |
|  |  | Patient deterioration | 187. After sifting = 0 |

| **Professional Bodies** | | | |
| --- | --- | --- | --- |
| **Date of Search: 09-11/03/2016** | | | |
| **Website/ Database** | **URL** | **Search Terms** | **Hits** |
| **Irish Association for Emergency Medicine (IAEM)** | [www.iaem.ie](http://www.iaem.ie/) | Single search box, no instructions. Boolean operators are accepted but only with certain search terms the more options added, the less it seems to work. Therefore, a manual search was performed. | 0 |
| **Royal College for Emergency Medicine (UK)** | [www.rcem.ac.uk](http://www.rcem.ac.uk/) | Single search box, no instructions. Boolean operators are accepted but only with certain search terms the more options added, the less it seems to work. Therefore, a manual search was performed. | 3 |
| **European Society for Emergency Medicine (EuSEM)** | [www.eusem.org](http://www.eusem.org/) | No results for simple searches such as: "patient deterioration", "track and trigger" or "early warning". | 0 |
| **American Academy of Emergency Medicine (AAEM)** | [www.aaem.org](http://www.aaem.org/) | (emergency OR trauma OR casualty OR triage) AND (care OR ward OR wards OR department OR departments OR unit OR units OR room OR rooms OR health OR healthcare OR hospital OR service) AND (warning OR trigger OR EWS OR MEWS OR POTTS) AND (deteriorate OR deterioration OR deteriorated OR deteriorates OR worse OR worsen OR worsening OR adverse OR weaken OR weakened OR weakens OR weaker OR “acute illness”) AND (Monitor or monitors or monitored or monitoring OR escalate OR escalates OR escalated OR escalation OR escalating OR reassess OR reassesses or reassessed or reassessment OR reassessing) AND (adult OR adults OR adulthood) | 67 Hits. After sifting = 0 |
| **American College of Emergency Physicians (ACEP)** | [www.acep.org](http://www.acep.org/) | Single search box, no instructions. Boolean operators not accepted. | 1 |
| **Society for Academic Emergency Medicine (SAEM)** | [www.saem.org](http://www.saem.org/) | (emergency OR trauma OR casualty OR triage) AND (care OR ward* OR department* OR unit* OR room* OR health* OR hospital OR service) AND (warning OR trigger OR EWS OR MEWS OR POTTS) AND (deteriorat* OR worse* OR adverse OR weaken* OR weaker OR “acute illness”) (Monitor* OR escalat* OR reassess*) AND (adult*) | 4. After sifting = 0 |
| **Canadian Association of Emergency Physicians** | [www.caep.ca](http://www.caep.ca/) | (emergency OR trauma OR casualty OR triage) AND (escalate* OR trigger OR warning OR ews OR potts) | 110. After sifting = 0 |
| **Australasian Society for Emergency Medicine (ASEM)** | [www.asem.org.au](http://www.asem.org.au/) | Manual^1^ | 0 |
| **Australasian College of Emergency Medicine (ACEM)** | [www.acem.org.au](http://www.acem.org.au/) | Manual | 1 |
| **International Federation for Emergency Medicine (IFEM)** | [http://www.ifem.cc](http://www.ifem.cc/) | Manual | 2 |
| **Faculty of Emergency Nursing** | [www.fen.uk.com](http://www.fen.uk.com/) | Manual | 0 |
| **RCN Emergency Care Association** | [www.rcn.org.uk](http://www.rcn.org.uk/) | Manual | 0 |
| **Emergency Nurses Association** | [www.ena.org](http://www.ena.org/) | Manual | 0 |
| **Canadian Emergency Nurses** | [www.nena.ca](http://www.nena.ca/) | Access to this site not allowed for security reasons |  |
| **European Society for Emergency Nursing** | [www.eusen.org](http://www.eusen.org/) | Manual | 0 |
| **Emergency Nursing weblinks** | [www.enw.org](http://www.enw.org/) | (emergency OR trauma OR casualty OR triage) AND (escalate OR trigger OR warning OR ews OR potts) | 5. After sifting = 0 |
| **Philippine Society of Emergency Care Nurses** | [www.philippinenursingdirectory.com/associations/philippine-society-of-emergency-care-nurses-psecn/](http://www.philippinenursingdirectory.com/associations/philippine-society-of-emergency-care-nurses-psecn/) | Manual | 3. After sifting = 0 |
| **Emergency Nursing Society of South Africa** | <http://emssa.org.za/enssa/> | Manual | 0 |
| **Australian College of Emergency Nursing** | [www.acen.com.au](http://www.acen.com.au/) | Manual | 0 |
| **College of Emergency Nursing Australia** | [www.cena.org.au](http://www.cena.org.au/) | Manual | 0 |
| **College of Emergency Nurses (New Zealand) CENNZ - NZNO** | [www.nzno.org.nz/colleges/college_of_emergency_nurses](http://www.nzno.org.nz/colleges/college_of_emergency_nurses) | Manual | 0 |
| **Hong Kong Emergency Nursing** | [www.hkena.org](http://www.hkena.org/) | Manual | 0 |

^1^Manual searches were performed on websites where regular electronic searching attempts were not useful. This is caused by reduced searching functionality such as search boxes that can only search one word, or a lack of search box. Manual searching involves exploring clickable webpage content e.g., tabs, buttons, hyperlinks etc. in an iterative way to identify relevant resources.

| **Grey Literature** | | | |
| --- | --- | --- | --- |
| **Date of Search: 12-13/03/2016** | | | |
| **Website/ Database** | **URL** | **Search Terms** | **Hits** |
| **RIAN** | <http://rian.ie/en/static/User#search> | All of 'patient', 'deterioration', 'emergency' and 'department' in all fields; with any of '"track and trigger"', '"early warning"', '', 'OR', 'EWS', 'OR', 'MEWS', 'OR' or 'POTTS' in all fields | 0 |
|  |  | All of 'patient', 'deterioration', 'emergency' and 'department' in all fields | 1 |
|  |  | All of 'early', 'warning', 'emergency' and 'department' in all fields | 0 |
|  |  | All of 'early' and 'warning' in all fields | 37. After sifting = 4 |
|  |  | All of 'track', 'and' and 'trigger' in all fields | 2. After sifting = 1 |
| **Proquest Dissertations and Theses UK & Ireland** | [www.library.nuigalway.ie](http://www.library.nuigalway.ie/) | ab(emergency OR trauma OR casualty OR triage) AND (care OR ward* OR department* OR unit* OR room* OR health* OR hospital OR service) AND (warning OR trigger OR EWS OR MEWS OR POTTS) AND (deteriorat* OR worse* OR adverse OR weaken* OR weaker OR "acute illness" OR Monitor* OR escalat* OR reassess*) AND adult* | 24 after sifting = 0 |
| **Proquest Dissertations and Theses A & I** | [www.library.nuigalway.ie](http://www.library.nuigalway.ie/) | ab(warning OR trigger OR EWS OR MEWS OR POTTS) AND ab(deteriorat* OR worse* OR adverse OR weaken* OR weaker OR "acute illness" OR Monitor* OR escalat* OR reassess*) AND adult* | 23. After sifting = 0 |
|  |  | ab(emergency OR trauma OR casualty OR triage) AND ab(care OR ward* OR department* OR unit* OR room* OR health* OR hospital OR service) AND ab(warning OR trigger OR EWS OR MEWS OR POTTS) AND (deteriorat* OR worse* OR adverse OR weaken* OR weaker OR "acute illness" OR Monitor* OR escalat* OR reassess*) AND adult* | 88. After sifting = 0 |

| **Clinical Trials Registries** | | | |
| --- | --- | --- | --- |
| **Date of Search: 12-13/03/2016** | | | |
| **Website/ Database** | **URL** | **Search Terms** | **Hits** |
| **CENTRAL** | [www.cochranelibrary.com](http://www.cochranelibrary.com/) | See Cochrane Library tab | n/a |
| **Prospero** | [www.crd.york.ac.uk/PROSPERO](http://www.crd.york.ac.uk/PROSPERO) | Track and trigger | 3. After sifting = 0 |
|  |  | Early warning | 12. After sifting = 2 |
|  |  | Patient deterioration emergency department | 0 |
|  |  | Patient deterioration emergency | 0 |
|  |  | Patient deterioration | 5. After sifting = 0 |
|  |  | EWS | 3. 1 relevant but already picked up in early warning search. 0 |
|  |  | MEWS | 6. 1 relevant but already picked up in early warning search. 0 |
|  |  | POTTS | 4. After sifting 0 |
| **ClinicalTrials.gov** | <https://clinicaltrials.gov/ct2/search/advanced> | "patient deterioration" AND (emergency OR trauma OR casualty OR triage) AND (warning OR trigger OR escalation OR EWS OR MEWS OR POTTS) Filters used: Adult (18–65) & Senior (66+) | 0 |
|  |  | patient deterioration AND (emergency OR trauma OR casualty OR triage) AND (warning OR trigger OR escalation OR EWS OR MEWS OR POTTS) Filters used: Adult (18–65) & Senior (66+) | 9. After sifting = 1 |
|  |  | (emergency department OR trauma OR casualty OR triage) AND (early warning OR track and trigger OR escalation OR EWS OR MEWS OR POTTS) Filters used: Adult (18–65) & Senior (66+) | 107. After sifting = 2 |
| **World Health Organization (WHO) International Clinical Trials Registry Platform (ICTRP)** | <http://apps.who.int/trialsearch/AdvSearch.aspx> | Advanced search strategy. Only options to limit to 1) title 2) condition or 3) intervention. Tried using following in intervention field: early warning or track and trigger or ews or mews or potts. | 1138 results. Skim through shows low specificity therefore strategy abandoned. |
|  |  | Advanced search option not useful. Basic search : (emergency OR trauma OR casualty OR triage) AND (care OR ward* OR department* OR unit* OR room* OR health* OR hospital OR service) AND (warning OR trigger OR escalat* OR EWS OR MEWS OR POTTS) AND (deteriorat* OR worse* OR adverse OR weaken* OR weaker OR “acute illness”) (Monitor* OR escalat* OR reassess*) AND (adult*) | Site unable to handle this strategy and kept crashing |
|  |  | Basic option used again: patient deterioration AND emegency department OR Triage AND early warning OR track and trigger OR EWS OR MEWS OR POTTS | 29. After sifting 9 were useful but overlap with Clinicaltrials.gov results. 6 kept |
