# Supplementary material for: The effectiveness of physiologically based early warning or track and trigger systems after triage in adult patients presenting to emergency departments: a systematic review
Source: BMC Emerg Med. 2017 Dec 6;17:38. doi: 10.1186/s12873-017-0148-z (PMC5719672; doi:10.1186/s12873-017-0148-z)
Supplement: Supplementary file 2 — Data Extraction. The elements that were extracted for each study type included in this review. (DOCX 33 kb) [file 12873_2017_148_MOESM2_ESM.docx]

**Additional File 2: Data Extraction**

| *Descriptive studies – types and use of systems* |
| --- |
| - Authors, time and country of study - Study aim and design - Number of participants and characteristics - Method(s) of data collection and analysis - Content (parameters) of the early warning system or TTS or scoring system, and escalation criteria - Findings on the use of early warning or track and trigger system(s) |
| *Descriptive studies – education programmes* |
| - Authors, time and country of study - Study aim and design - Number of participants and characteristics - Method(s) of data collection and analysis - Content (parameters) of the early warning system or TTS or scoring system, and escalation criteria - Information on the educational programme or communication tool - Findings on the use of educational programme or communication tool concerning an early warning system or TTS or scoring system |
| *Guidelines* |
| - Guideline team (including qualifications), time and country of guideline - Guideline development strategy - Scope - Key recommendations - Implementation strategy - Audit strategy |
| *Effectiveness studies* |
| - Authors, time and country of study - Study aim and design - Number of participants and characteristics - Method(s) of data collection and analysis - Intervention (content (parameters) of the early warning system or TTS or scoring system, and escalation criteria) and control - Outcomes - Findings, including effect estimates |
| *Development and validation studies* |
| - Authors, time and country of study - Study aim and design - Number of participants and characteristics - Method(s) of data collection and analysis - Content (parameters) of the early warning system or TTS or scoring system, and escalation criteria - Reference criteria (outcomes) - Findings, including predictive ability measures |
| *Health economics* |
| - Authors, time and country of study - Study aim and design - Number of participants and characteristics - Method(s) of data collection and analysis - Measures of cost - Outcomes |
