# Supplementary material for: The effectiveness of physiologically based early warning or track and trigger systems after triage in adult patients presenting to emergency departments: a systematic review
Source: BMC Emerg Med. 2017 Dec 6;17:38. doi: 10.1186/s12873-017-0148-z (PMC5719672; doi:10.1186/s12873-017-0148-z)
Supplement: Supplementary file 3 — Risk of bias and methodological quality assessment. A detailed description of the risk of bias/quality assessment of the included studies. (DOCX 53 kb) [file 12873_2017_148_MOESM3_ESM.docx]

**Additional File 3: Risk of bias and methodological quality assessment**

**Descriptive studies: Extent of use**

**Assessed using the Quality Assessment Tool adapted from NIH (2014)^23^**

Note: the studies shown here relate to table 4 of the evidence tables.

| **Criteria** | **Australian Commission (2011)^21^** | **Considine et al (2012)^19^** | **Coughlan et al (2015)^22^**  Conference abstract | **Griffiths et al (2012)^20^** |
| --- | --- | --- | --- | --- |
| 1. Was the research question or objective in this paper clearly stated? | Yes | Yes | Yes | Yes |
| 2. Was the study population clearly specified and defined? | Yes | Yes | No | Yes |
| 3. Was the participation rate at least 50%? | Yes | No  (Total sample represented 9.8% of ED EWS activations) | NR | Yes  (57% response rate) |
| 4. Were all the subjects selected or recruited from the same or similar populations (including the same time period)? Were inclusion and exclusion criteria for being in the study pre-specified and applied uniformly to all participants? | Yes | Yes | NR | NR |
| 5. Was a sample size justified? | No | No | NR | No |
| 6. Were the outcome measures clearly defined, valid, reliable, and implemented consistently across all study participants? | NR | Yes | NR | Yes |
| **Quality Rating (Good, Fair, or Poor)** | **Fair** | **Fair** | **Poor** | **Fair** |

*CD, cannot determine; NA, not applicable; NR, not reported

**Descriptive studies: Compliance**

**Assessed using the Quality Assessment Tool for Before-After (Pre-Post) Studies with No Control Group (NIH 2014)^23^**

Note: the studies shown here relate to table 5 of the evidence tables.

| **Criteria** | **Austen et al (2012)^25^** | **Christensen et al (2011)^23^** | **Hudson et al (2015)^26^** | **Johnson et al (2014)^24^** | **Wilson et al (2013)^27^** |
| --- | --- | --- | --- | --- | --- |
| 1. Was the study question or objective clearly stated? | Yes | Yes | Yes | Yes | Yes |
| 2. Were eligibility/selection criteria for the study population pre-specified and clearly described? | Yes | Yes | No | Yes | Yes |
| 3. Were the participants in the study representative of those who would be eligible for the test/service/intervention in the general or clinical population of interest? | Yes | Yes | Yes | Yes | Yes |
| 4. Were all eligible participants that met the pre-specified entry criteria enrolled? | Yes | Yes | CD | Yes | No  (Restricted to times when a member of research team was on duty) |
| 5. Was the sample size sufficiently large to provide confidence in the findings? | CD | Yes | CD | Yes | No |
| 6. Was the test/service/intervention clearly described and delivered consistently across the study population? | Yes | Yes | Yes  (post) | N/A | Yes |
| 7. Were the outcome measures pre-specified, clearly defined, valid, reliable, and assessed consistently across all study participants? | Yes | Yes | No  (not pre-specified) | Yes | Yes |
| 8. Were the people assessing the outcomes blinded to the participants' exposures/interventions? | N/A | N/A | NR | N/A | N/A |
| 9. Was the loss to follow-up after baseline 20% or less? Were those lost to follow-up accounted for in the analysis? | Yes | Yes | Yes | Yes | Yes |
| 10. Did the statistical methods examine changes in outcome measures from before to after the intervention? Were statistical tests done that provided p values for the pre-to-post changes? | N/A | N/A | Yes | N/A | N/A |
| 11. Were outcome measures of interest taken multiple times before the intervention and multiple times after the intervention (i.e., did they use an interrupted time-series design)? | N/A | N/A | N/A | N/A | N/A |
| 12. If the intervention was conducted at a group level (e.g., a whole hospital, a community, etc.) did the statistical analysis take into account the use of individual-level data to determine effects at the group level? | N/A | N/A | N/A | N/A | N/A |
| **Quality Rating (Good, Fair, or Poor)** | **Good** | **Good** | **Fair** | **Good** | **Fair** |

*CD, cannot determine; NA, not applicable; NR, not reported

**Effectiveness studies:**

**Assessed using the EPOC Risk of Bias Tool^27^**

Note: the studies shown here relate to table 7 of the evidence tables.

| **EPOC criteria** | **Shuk-Ngor et al (2015)^28^**  **Two group non-randomised comparison**  **(MEWS group versus usual observation group)** |
| --- | --- |
| Was the allocation sequence adequately generated? | High risk of bias |
| Was the allocation adequately concealed? | High risk of bias |
| Were baseline outcome measurements similar? | Unclear risk of bias |
| Were baseline characteristics similar? | Low risk of bias |
| Were incomplete outcome data adequately addressed? | Low risk of bias |
| Was knowledge of the allocated interventions adequately prevented during the study? | Unclear risk of bias |
| Was the study adequately protected against contamination? | Unclear risk of bias |
| Was the study free from selective outcome reporting? | Low risk of bias |
| Was the study free from other risks of bias? | Unclear risk of bias |
| **Overall Risk of Bias Judgement** | **High risk of bias** |

**Effectiveness studies:**

**Assessed using the GRADE assessment of quality of evidence^26^**

Note: the studies shown here relate to table 7 of the evidence tables.

| **Study: Shuk-Ngor et al (2015)^28^** | | | | | | | | | | | | | | | |
| --- | --- | --- | --- | --- | --- | --- | --- | --- | --- | --- | --- | --- | --- | --- | --- |
| **Outcome** | **No. of studies** | **Design** | **ROB** | **Inconsistency** | **Indirectness** | **Imprecision** | **Publication bias** | **Large magnitude effect** | **Dose-response gradient** | **Effect of plausible residual confounding** | **MEWS** | **Usual observation** | **Relative risk** | **Quality** |  |
| Change in management (no of activations) | 1 | Non-RCT | Serious ROB^a^ | No serious inconsistency | No serious indirectness | Serious imprecision^b^ | No serious publication bias | No large effect | N/A | N/A | 1/10 of 269 | 1/20 of 275 | 2.0 (95% CI 1.1; 3.8) | Very low |  |
| Adverse events | 1 | Non-RCT | Serious ROB^a^ | No serious inconsistency | No serious indirectness | Serious imprecision^b^ | No serious publication bias | No large effect | N/A | N/A | 1/269 | 1/275 | 1.02 (95% CI 0.06; 16.3) | Very low |  |
| ^a^Downgraded one level because the ROB for this study was rated as high.  ^b^Downgraded one level because of small number of events in the study. | | | | | | | | | | | | | | | |

**Development and validation studies – Scoping Review:**

**Assessed using the Quality Assessment Tool adapted from NIH (2014)^23^**

Note: the studies shown here relate to table 9 of the evidence tables.

| **Criteria (Quality Assessment Tool adapted from NIH (2014))** | **Challen et al (2011)^64^**  A Scoping review |
| --- | --- |
| 1. Was the research question or objective in this paper clearly stated? | Yes |
| 2. Was the study population clearly specified and defined? | N/A |
| 3. Was the participation rate at least 50%? | N/A |
| 4. Were all the subjects selected or recruited from the same or similar populations (including the same time period)? Were inclusion and exclusion criteria for being in the study pre-specified and applied uniformly to all participants? | Yes |
| 5. Was a sample size justified? | N/A |
| 6. Were the outcome measures (dependent variables) clearly defined, valid, reliable, and implemented consistently across all study participants? | Yes |
| **Quality Rating (Good, Fair, or Poor)** | **Good** |

**Development and validation studies:**

**Assessed using the Quality Assessment Tool (adapted from Kansagara *et al* 2011)**

Note: the studies shown here relate to tables 10, 11 and 12 of the evidence tables and are presented alphabetically by author.

| **Study (year)** | **Adequate description of population^a^** | **Non-biased selection^b^** | **Adequate prognostic factor measurement^c^** | **Adequate outcome measurement^d^** | **Method of validation^e^** | **Overall risk of bias** |
| --- | --- | --- | --- | --- | --- | --- |
| Albright et al (2014)^29^ (Development & Validation) | Yes | No  (Only those who had blood cultures or influenza swab were included) | Yes  (SOS (for high risk of outcome) ≥ 6) | Yes | Yes  (New instrument against other ED system; AUROC and SOS ≥ 6 versus < 6) | Low risk of bias |
| Alam et al (2015)^53^ (Validation) | Yes | No  (Recruitment between 12-8pm only) | Yes  (NEWS 0-4, 5-6, ≥7) | Yes | Unclear  (AUROC reported for only 2 of 4 outcomes) | Unclear risk of bias |
| Armagan et al (2008)^54^ (Validation) | Yes | Unclear  (No clear statement) | Yes  (mEWS > 4) | Yes | Unclear  (Multi-variate regression only) | Unclear risk of bias |
| Bulut et al (2014)^37^ (Validation) | Yes | Unclear  (No clear statement) | Yes  (MEWS ≥ 5  REMS > 13) | Yes | Yes  (Against other system; AUROC) | Low risk of bias |
| Burch et al (2008)^63^ (Validation) | Yes | No  (Every 6^th^ day only and 790 patients included = 70.2% of potential study cohort) | Unclear  (Indicates MEWS ≥ 5 but no clear statement of cut-off for high risk of outcome) | Yes | Unclear  (Univariate regression only) | High risk of bias |
| Cattermole et al (2009)^31^ (Development & Validation) | Yes | Yes | Unclear  (No clear statement of cut-off scores for systems) | Yes | Yes  Against other systems; multivariate regression and AUROC | Low risk of bias |
| Cattermole et al (2014)^35^ (Development & Validation) | Yes | No  (Week days only) | Unclear  (No clear statement of cut-off scores for systems) | Yes | Yes  (Against other systems; multivariate regression and AUROC) | Unclear risk of bias |
| Christensen et al (2011)^30^ (Development & Validation) | Yes | Yes | Yes  BEWS ≥ 5 | Yes | Yes  (BEWS ≥ 5 versus BEWS < 5; sensitivity, specificity) | Low risk of bias |
| Cıldır et al (2013)^38^ (Validation) | Yes | Unclear  (No clear statement) | Yes  (Sepsis defined  MEWS > 4  CCI > 4) | Yes | Yes  (Against other systems; survivor versus non-survivor and AUROC) | Low risk of bias |
| Considine et al (2015)^39^  (Validation) | Yes | Yes  (stratified random sample) | Yes  (Single parameter system) | Yes | No  (Only Mann-Whitney U and Kruskal Wallis tests) | Low risk of bias |
| Corfield et al (2014)^74^ (Validation) | Yes | Yes | Yes  (N/A for high risk prediction cut-off scores as development study) | Yes | Yes  (Regression; survivor versus non-survivor and AUROC) | Low risk of bias |
| Correia et al (2014)^55^ (Validation | Yes | Yes | Unclear (No clear description of the score) | Yes | No (Only t-test and chi square test) | Unclear risk of bias |
| Dundar et al (2015)^41^ (Validation) | Yes | Yes | Yes  (optimal cut-off determined by Youden’s index) | Yes | Yes  (Two systems compared and AUROC) | Low risk of bias |
| Eick et al (2015)^42^ (Validation) | Yes | Yes | Unclear  Cut-offs for DC and MEWS (high-risk) not clearly provided | Yes | Yes  (Against other systems; multivariate regression; bootstrapping and AUROC) | Low risk of bias |
| Geier et al (2013)^32^ (Development & Validation) | Yes | Yes | Yes  (ESI ≤ 2; MEWS ≥ 5  MEDS ≥ 8; CCI ≥ 2) | Yes | Yes  (Against other systems and AUROC) | Low risk of bias |
| Graham et al (2007)^56^ (Conference abstract only) (Validation) | Yes | Yes | Unclear  (Insufficient detail) | Yes | Unclear  (Mentions AUROC but insufficient detail to fully assess) | Unclear risk of bias |
| Gu et al (2015)^18^ (Only abstract in English) (Validation) | Unclear  Partial details given in results but no clear statement on selection criteria | Unclear  No clear statement | Yes  (MEWS ≥ 5) | Yes | Yes  (Multivariate regression and MEWS positive (≥ 5) versus MEWS negative (0-4)) | Unclear risk of bias |
| Heitz et al (2010)^43^ (Validation) | Yes | Yes | Yes  (N/A for high risk prediction cut-off scores as development study) | Yes | Yes  (Multivariate regression; AUROC and MEWS Max cut-of scores (≥ 1 through to ≥ 9)) | Low risk of bias |
| Ho et al (2013)^44^ (Validation) | Yes | No  (No clear statement and 8am to 6pm recruitment only) | Yes  (MEWS ≥ 4) | Yes | Yes  (Regression; AUROC and MEWS < 4 versus MEWS ≥ 4) | Low risk of bias |
| Hock Ong et al (2012)^57^ (Validation) | Yes | No  (No clear statement and ‘office hours’ recruitment only) | Unclear  (Cut-offs for DC and MEWS (high-risk) not clearly provided) | Yes | Yes  (MEWS versus ML system and AUROC) | Unclear risk of bias |
| Howell et al (2007)^45^ (Validation) | Yes | Yes | Unclear  (Refers to other publication for calculations) | Yes | Yes  (Against other systems; regression and AUROC) | Low risk of bias |
| Jo et al (2013)^46^ (Validation) | Yes | Yes | Unclear  (Cut-off for VIEWS-L not clearly provided) | Yes | Yes (Against other system; AUROC) | Low risk of bias |
| Jo et al (2016)^47^ (Validation) | Yes | Unclear | Yes (Optimal cut-off determined by Youden Index) | Yes | Yes  (Against other systems; AUROC) | Low risk of bias |
| Jones et al (2005)^48^ (Validation) | Yes | Unclear  (No clear statement) | Yes  (Predicted mortality > 50%) | Yes | Yes  (Against other systems; AUROC) | Low risk of bias |
| Junhasavasdikul et al (2013)^58^ (Validation) | Yes | Unclear  (States all but no clear statement if consecutive enrolment occurred ) | Unclear  (Vital sign cut-off values for high-risk not provided) | Yes | Yes  (Multivariate regression and R^2^ ) | Unclear risk of bias |
| Keep et al (2016)^49^ (Validation) | Yes | Yes | Unclear  (Cut-off values for high-risk not provided) | Yes | Yes  (NEWS cut-off ≥ 1 through to ≥ 11; AUROC) | Low risk of bias |
| Liu et al (2014)^36^ (Development & Validation) | Yes | Unclear  (No clear statement) | Unclear  (No detail on scores) | Yes | Yes  (Against other systems and AUROC) | Unclear risk of bias |
| Naidoo et al (2014)^62^ (Validation) | No  (Selection criteria unclear) | No  (Every 5^th^ record) | Yes  (TEWS ≥ 7) | Yes | Unclear  (TEWS < 7 versus TEWS ≥ 7 only) | High risk of bias |
| Nguyen et al (2012)^59^ (Validation) | Yes | Unclear  (Only patients enrolled in the registry for whom all 3 physiologic scores were available) | Yes | No  (Reports mortality but it is not pre-specified or defined) | Yes  (Against other systems; AUROC) | Unclear risk of bias |
| Olsson & Lind (2003)^33^ (Development & Validation) | Yes | Yes | Unclear  (No clear statement on cut-off scores for high risk) | Yes | Yes  (Split sample technique; multivariate regression; AUROC) | Low risk of bias |
| Olsson et al (2004)^34^ (Development & Validation) | Yes | Yes | Unclear  (No clear statement on cut-off scores for high risk) | Yes | Yes  (Split sample technique; multivariate regression and AUROC) | Low risk of bias |
| Subbe et al (2006)^50^ (Validation) | Yes | Yes | Yes  (Cut-off scores for risk prediction provided for each system) | Unclear  (ICU admissions reported but outcome not pre-specified or defined) | Yes  (Against other systems  (correlations)) | Low risk of bias |
| Vorwerk et al (2009)^51^ (Validation) | Yes | Yes | Yes  (Cut-off scores for risk prediction provided for each system) | Yes | Yes  (By outcome groups and AUROC) | Low risk of bias |
| Wang et al (2016)^60^ (Validation) | Yes | Unclear  (Only 99 of 234 initially eligible had a peri-arrest MEWS) | Unclear  (No clear statement on cut-off scores for high risk) | Yes | Unclear  (Multivariate regression only) | Unclear risk of bias |
| Williams et al (2016)^52^ (Validation) | Yes | Yes | Yes  (Table 1; score > 15 = 50% predicted mortality) | Yes | Yes  (Against other systems and AUROC) | Low risk of bias |
| Wilson et al (2016)^61^ (Validation) | Unclear  (Selection criteria unclear) | No  (Recruitment restricted to times when research team available) | Unclear  (Cut-off values not provided) | Yes | Unclear | High risk of bias |

^a^Study describes inclusion criteria for selecting patients, and for enrolled patients describes duration and severity of symptoms, demographics (at least age), and setting (primary care vs. occupational vs. other).

^b^Study either reports enrolling (or attempting to enrol) a consecutive series of patients meeting inclusion criteria, or a random sample.

^c^Study describes reproducible and appropriate methods for measuring prognostic factors

^d^Study describes reproducible and appropriate methods to define and identify outcome

^e^Method of validation is clear and appears to be appropriate
