# Supplementary material for: The effectiveness of physiologically based early warning or track and trigger systems after triage in adult patients presenting to emergency departments: a systematic review
Source: BMC Emerg Med. 2017 Dec 6;17:38. doi: 10.1186/s12873-017-0148-z (PMC5719672; doi:10.1186/s12873-017-0148-z)
Supplement: Supplementary file 4 — Development and validation studies, additional information. In depth study information on the included studies developing and/or validating early warning system(s). (DOCX 69 kb) [file 12873_2017_148_MOESM4_ESM.docx]

**Additional File 4: Development and validation studies, additional information**

***Table 7. Evidence table: Development and validation studies – Patient groups differentiated by triage category***

| **Authors (year), country** | **Study aim** | **Study design (type)** | **Participants** | **Content of system/tool** | **Reference criteria** | **Results** |
| --- | --- | --- | --- | --- | --- | --- |
| Alam et al (2015),^53^ the Netherlands  **Risk of bias:** Unclear | To explore the performance of NEWS with regard to predicting adverse outcomes in adult patients and the ability of NEWS to predict the need for hospital admission in an ED population. | Prospective cohort (Validation) | 274 patients (≥18 years) presenting (T0) to the ED of an urban academic tertiary care centre between 7 Jan-15 Feb 2013 with an Emergency Severity Index score of 2 and 3 not triaged to the resuscitation room. For 247 of these 274 patients, the NEWS was calculated an hour later (T1). Only 133 of the 247 patients could be followed up at discharge from the ED (T2). | **NEWS**: Parameters: Respiratory rate, SBP, HR, temperature, oxygen saturation | Hospital admission, length of stay, ICU admission, mortality | **Hospital admission** (n=130)  NEWS significantly associated with admission at all 3 time points (p < 0.001).  • T0: AUROC 0.66 (95% CI 0.60–0.73)  • T1: AUROC 0.69 (95% CI 0.62–0.75)  • T2: AUROC 0.70 (95% CI 0.61–0.79)  **Length of stay**  NEWS significantly associated with length of stay at all 3 time points (p < 0.001).  Median length of stay more than doubled for a score >7 compared with a score of 0–4. (AUROC not provided)  **ICU admission** (n=10)  NEWS significantly associated with ICU admission at all 3 time points (T0: p=0.003; T1: p=0.001; T2: p=0.046). (AUROC not provided)  **30-day Mortality** (n=11)  NEWS significantly associated with mortality at all 3 time points (p < 0.001).  30-day mortality was not significantly related to ESI (p = 0.816).  • T0: AUROC 0.77 (95% CI 0.62–0.92)  • T1: AUROC 0.87 (95% CI 0.77–0.96)  • T2: AUROC 0.77 (95% CI 0.57–0.97).  Of the individual physiological measures of NEWS:  • Respiratory rate was associated with mortality at all measured time points (T0: p=0.017; T1: p<0.001; T2: p=0.014).  • Pulse rate had a strong correlation with mortality at T1 (p=0.037)  No correlations could be found for all other physiological parameters. |
| Armagan et al (2008),^54^ Turkey  **Risk of bias**: Unclear | To determine the predictive validity of the Modified Early Warning Score (MEWS) in a Turkish ED setting. | Prospective cohort (Validation) | 309 patients (Triage I, II, II) in ED of one hospital between April-Aug 2007. | **MEWS:** Parameters: SBP, pulse rate, respiratory rate, temperature, AVPU score | Hospital admission, ICU admission, in-hospital death, ED death | Low risk (MEWS≤4) (n=106); high risk (MEWS >4) (n=203) MEWS (cut-off >4) • Admission to hospital: adjusted OR 1.56 (95% CI 0.93-2.98) • Admission to ICU: adjusted OR 1.95 (95% CI 1.04-366.00) (p=0.04) • Death in ED: adjusted OR 35.13 (95% CI 4.58-269.40) (p<0.001) • Death in hospital: adjusted OR 14.80 (95% CI 5.52-39.70) (p<0.001) |
| Bulut et al (2014),^37^ Turkey  **Risk of bias**: Low | Compare the efficacy of Modified Early Warning Score (MEWS) and Rapid Emergency Medicine Score (REMS) on in-hospital mortality, and as a predictor of hospitalisation in general medical and surgical patients admitted to ED. | Prospective, multicentre cohort (Validation) | 2000 general medical & surgical patients (red & yellow triage category) presenting to EDs of 3 hospitals between Oct 2011-April 2012. | **REMS:** Parameters: Age, HR, Temp, Respiratory Rate, Mean arterial pressure, GCS, oxygen saturations **MEWS:** Parameters: SBP, HR, Respiratory rate, Temp, AVPU | Admission to ward or ICU/HDU, in-hospital mortality | Median (range):  • MEWS: 1 (0-9); REMS: 5 (0-16)  40.8% hospitalised ward, 29.8%-ICU/HDU, 29.2% discharged.  Total in-hospital mortality was 7.7% (n=153).  **Predicting in-hospital mortality** • REMS (6–13) vs REMS <6: RR 2.92 (95% CI 0.03 to 4.22); p<0.001 • REMS (>13) vs REMS <6: RR 14.56 (95% CI 4.57 to 46.57); p<0.001 • MEWS≥5 vs MEWS <5: RR 3.84 (95% CI 2.36 to 6.24); p<0.001 • MEWS AUROC: 0.63 (95% CI 0.61-0.65)  • REMS AUROC: 0.71 (95% CI 0.67-0.72)  • Performance of REMS was higher (p<0.001)  **Predicting discharge vs hospitalisation:** • MEWS AUROC: 0.57 (95% CI 0.55-0.59)  • REMS AUROC: 0.64 (95% CI 0.62-0.66)  • Performance of REMS was higher (p<0.001)  **Predicting admission to ICU/HDU:** • MEWS AUROC: 0.54 (95% CI 0.52-0.56) • REMS AUROC: 0.59 (95% CI 0.57 to 0.61) • Performance of REMS was higher (p<0.001) |
| Cattermole et al (2009),^31^ Hong Kong  **Risk of bias**: Low | (1) Determine a new prognostic score making use of rapidly available and easily measurable physiological parameters and initial laboratory tests in resuscitation room patients, in order to identify patients most at risk of death or in need of ICU care. (2) To compare the new score with APACHE II, RTS, REMS and MEWS scores | Prospective cohort (Development & Validation) | 330 ED patients ≥18 years of age triaged to resuscitation room in 1 hospital between 9th April & 6th May 2006. | **Prince of Wales ED Score (PEDS)**  (new score)  Parameters: SBP, GCS, Glucose, HCO_3_, white blood cells, metastatic cancer history  **Revised Trauma Score** (RTS) **Rapid Emergency Medicine Score** (REMS)  MEWS  **Acute Physiology and Chronic Health Evaluation score** (APACHE II)  Parameters not stated in this report | **Primary outcome**: death admission to ICU within 7 days of ED attendance (vs survival at 7 days without ICU admission)  **Secondary outcome measures:** 30 day mortality and hospital length of stay. | Poor outcome: 23.0% (77/330) (40 (12.1%) admitted to ICU or 41 (12.4%) died within 7 days). PEDS score ranged from -2 to 58.  **Comparison of PEDS, APACHE II, RTS, REMS and MEWS (n=234) for the primary outcome:**  PEDS: AUROC 0.90 (0.87–0.94), Sensitivity 0.87, Specificity 0.80, PPV 0.57, NPV 0.95, Accuracy 0.82 APACHE II:  AUROC 0.73 (0.68–0.78), Sensitivity 0.61, Specificity 0.70, PPV 0.44, NPV 0.86 Accuracy 0.73 RTS: AUROC 0.75 (0.70–0.79), Sensitivity 0.58, Specificity 0.83, PPV 0.51, NPV 0.87 Accuracy 0.82 REMS:  AUROC 0.70 (0.64–0.75), Sensitivity 0.51, Specificity 0.79, PPV 0.42, NPV 0.84 Accuracy 0.73 MEWS: AUROC 0.76 (0.71–0.81), Sensitivity 0.69, Specificity 0.74, PPV 0.45, NPV 0.89 Accuracy 0.73  **Comparison of PEDS, APACHE II, RTS, REMS and MEWS (n=234) for the secondary outcome (30 day mortality):**  PEDS: AUROC 0.90 (0.86–0.93)  APACHE II:  AUROC 0.84 (0.79–0.88) RTS: AUROC 0.77 (0.72–0.81) REMS:  AUROC 0.77 (0.72–0.82) MEWS: AUROC 0.75 (0.70–0.80) |
| Cattermole et al (2013),^35^ Hong Kong  **Risk of bias**: Unclear | (1) To validate Prince of Wales ED Score (PEDS) in comparison with other prognostic scores (MEWS, SCS, REMS, MEES, MEDS, Worthing & NEWS); (2) to simplify and refine the score, using only variables that are immediately available in the resuscitation room; (3) to validate the new score to devise original PEDS | Prospective cohort (Development & Validation) | 234 consecutive, ≥18 years of age, patients managed in resuscitation room during weekdays over a 3 month period. | **The Resuscitation Management score** (THERM) (refined PEDS score): Parameters: GCS, HCO_3_^−^ and SBP  **PEDS** Parameters: SBP, GCS, Glucose, HCO_3_, white blood cells, metastatic cancer history  **MEWS Simple Clinical Score** (SCS) **REMS  Mainz Emergency Evaluation Score** (MEES)  **National Early Warning Score** (NEWS) **Worthing** (excluded because it is a condition specific score) **Mortality in the ED Sepsis** (MEDS) (excluded because it is a condition specific score) Parameters not stated in report. | **Primary outcome**: death or admission to ICU.  **Secondary outcomes**: 30 day mortality & hospital length of stay. | 37/234 admitted to ICU or died within 7 days.  PEDS: AUROC 0.75 (95% CI 0.69 to 0.80) MEES: AUROC 0.75 (95% CI 0.69 to 0.80) MEWS: AUROC 0.73 (95% CI 0.67 to 0.79) NEWS: AUROC 0.71 (95% CI 0.64 to 0.76) REMS: AUROC 0.70 (95% CI 0.64 to 0.76) SCS: AUROC 0.70 (95% CI 0.64 to 0.76)  THERM scores: max score=37, High risk <30, medium risk (30.1-35), low risk (35.1-37).  **Comparison of THERM and NEWS (n=234)**  THERM:  AUROC: 0.84 (0.79 to 0.88) High risk cut-off: Sensitivity 0.57 (0.40 to 0.73), Specificity 0.89 (0.84 to 0.93), PPV 0.50 (0.34 to 0.66), NPV 0.92 (0.87 to 0.95) Medium risk cut-off: Sensitivity 0.89 (0.75 to 0.97), Specificity 0.65 (0.58 to 0.72), PPV 0.32 (0.23 to 0.42), NPV 0.97 (0.92 to 0.99)  NEWS:  AUROC: 0.71 (0.64 to 0.76) High risk cut-off: Sensitivity 0.65 (0.48 to 0.80), Specificity 0.71 (0.64 to 0.77), PPV 0.29 (0.20 to 0.40), NPV 0.91 (0.86 to 0.95) Medium risk cut-off: Sensitivity 0.92 (0.78 to 0.98), Specificity 0.44 (0.37 to 0.51), PPV 0.24 (0.17 to 0.31), NPV 0.97 (0.91 to 0.99)  THERM had superior specificity; there was no significant difference in AUROC, sensitivity or predictive values. |
| Christensen et al (2011),^30^ Denmark  **Risk of bias**: Low | To evaluate ability of the Bispebjerg EWS (BEWS) to identify critically ill patients in ED and to examine feasibility of using BEWS to activate Medical Team response | Retrospective cohort (Development & Validation) | Random sample of 300 'red' category patients visiting ED of 1 hospital between April-Sept 2009. | **BEWS** Parameters: Respiratory Rate, HR, SBP, Temp, LOC | Admission to ICU within 48 hrs of arrival at ED or death within 48hrs of arrival at ED. | 138 patients out of 300 were excluded for insufficient data; 162 included.  Activated Emergency Call (EC): 24  Admitted to ICU within 48 hrs of ED: 4 (2 died)  Deaths within 48hrs ED: 6  BEWS ≥ 5 (vs <5): **Death within 48 hours of arrival:**  • RR 20.3 (95% CI 6.9-60.1) • Sensitivity 83.0%, Specificity 83.0%, PPV 16.0, NPV 99.0 **ICU admission within 48 hours of arrival:**  • RR 4.1 (95% CI 1.5- 10.9)  • Sensitivity 50.0%, Specificity 81.0%, PPV 6.0, NPV 98.0 **Critically ill:**  • RR 6.8 (95% CI 3.3-13.8) • Sensitivity 63.0%, Specificity 82.0%, PPV 16.0, NPV 98.0 |
| Gu et al (2015),^18^ China  **Risk of bias:** Unclear (Only abstract in English) | To evaluate the value of Modified Early Warning Score (MEWS) in predicting mortality of critically ill patients admitted to the emergency department. | Prospective cohort (Validation) | 176 emergency patients admitted to resuscitation room of one hospital between 13 Feb-20 April 2014. | **MEWS** (parameters not listed in abstract) | 3-day mortality, all deaths, and composite outcome of intensive care unit (ICU) transfer, cardio-pulmonary resuscitation, and death. | Mean MEWS 4.30±2.74; 74 cases MEWS ≥ 5 and 102 in MEWS 0-4.  **3-days mortality (n=41)** MEWS 0-1 (12.7% (13/102); ref) MEWS ≥ 5 (37.8 (28/74); OR 4.2 (95%CI 2.0 - 8.8, P < 0.001))  Multi-regression logistic showed abnormal mental status (OR 3.6, 95% CI = 1.5-8.4, P = 0.003) but not MEWS ≥ 5 (OR = 1.7, 95%CI = 0.6-4.5, P = 0.3) was the predictor of 3-day mortality.  **All death (n=58)**  MEWS 0-1 (17.7% (18/102); ref) MEWS ≥ 5 (54.1 (40/74); OR 5.5 (95%CI 2.8- 10.9, P < 0.001))  **ICU transfer, cardio-pulmonary resuscitation and death (n=74)** MEWS 0-1 (25.5% (26/102); ref) MEWS ≥ 5 (64.9 (48/74); OR 5.4 (95%CI 2.8- 10.4, P < 0.001)) |
| Ho et al (2013),^44^ Singapore  **Risk of bias**: Low | To validate the use of the Modified Early Warning Score (MEWS) as a predictor of patient mortality and intensive care unit (ICU)/ high dependency unit (HDU) admission in an Asian population. | Retrospective cohort (Validation) | 1024 critically ill patients, ≥18 years of age, presenting to a large Asian tertiary ED between Nov 2006 and Dec 2007, and requiring continuous ECG monitoring and Patient Acuity category Scale (PACS) of 1 or 2. | **MEWS:** Parameters: SBP, pulse rate, respiratory rate, temperature, AVPU score | Mortality during the inpatient period following admission from the ED up to 30 days and direct admission from the ED to the high dependency unit, intermediary care area or the intensive care unit. | 713 patients MEWS score <4; 311 patients MEWS ≥4.  **Mortality** 47 deaths (6.6%) in MEWS <4; 53 (17.0%) deaths in MEWS ≥4 (p<0.001)  For cut-off value ≥4: Sensitivity: 47.0 Specificity: 27.9 PPV: 6.7 NPV: 83.0 AUROC: 0.68  **Admission** 267 (37.4%) were admitted to HDU/ICA in MEWS <4; 86 (27.7%) admitted to HDU/ICA in MEWS ≥4 (p=0.00).  For cut-off value of 4: Sensitivity: 74.2 Specificity: 33.9 PPV: 46.7 NPV: 62.7 AUROC: 0.5 |
| Hock Ong et al (2012),^57^ Singapore  **Risk of bias**: Unclear | To validate a novel Machine Learning (ML) score incorporating Heart Rate Variability (HRV) for risk stratification of critically ill patients presenting to the ED by comparing the area under the curve, sensitivity and specificity for prediction of cardiac arrest with the Modified Early Warning Score (MEWS). | Prospective cohort (Validation) | 925 patients, ≥18 years of age, requiring continuous ECG monitoring triaged as Patient Acuity Category Scale (PACS) 1 or PACS 2 were eligible. | **MEWS:** Parameters: SBP, pulse rate, respiratory rate, temperature, AVPU score **ML-based score**: (excluded because HRV is not a routine measure) | Cardiac arrest within 72 hours of presentation to the ED, death after admission (in-hospital death during current admission, including within 72 hours). | 4.6% (43) developed cardiac arrest within 72 hours; 9.3% (86) died after admission.  **Cardiac arrest**  **MEWS** Sensitivity: 74.4 Specificity: 54.2 PPV: 7.4 (5.3-10.3) NPV: 97.8 (95.9-98.8) +LR: 1.6 (1.3-2.0) AUROC: 0.7  **Death after admission**  **MEWS** Sensitivity: 74.4 Specificity: 55.7 PPV: 14.7 (11.5-18.4) NPV: 95.5 (93.2-97.1) +LR: 1.7 (1.5-1.9) AUROC: 0.7 |
| Keep et al (2015),^49^ UK  **Risk of bias**: Low | Explore relationship between initial National Early Warning Score (NEWS UK) in ED and diagnosis of Severe Sepsis (SS) | Retrospective cohort (Validation) | 500 patients, >16 years of age, presenting at ED of an urban hospital in a 5-day period, with triage category 1-3 (Manchester Triage Score). | **NEWS:** Parameters: Respiratory Rate, HR, O_2_ saturations, SBP, Temp, LOC. | Septic shock (SS) | Sepsis: 9.9% (n=50); Septic shock (SS): 5.4% (n=27)   **Prediction of SS:** NEWS optimal cut-off ≥3: AUC 0.90 (95% CI 0.84-0.94). Sensitivity 92.6% (95% CI, 74.2%-98.7%), specificity of 77% (95% CI 72.8% to 80.6%), PPV 18.7% (95% CI 12.7% to 26.5%), NPV 99.5% (95%CI 97.8%-99.9%) |
| Lui et al (2014),^36^ Singapore  **Risk of bias**: Unclear | To propose an intelligent scoring system and explore the utility of combining Heart Rate Variability (HRV) and 12-lead ECG parameters, and vital signs to predict acute cardiac complications within 72 h. | Prospective cohort (Development & Validation) | 564 (of eligible 702) chest pain patients aged ≥30, triaged as PACS 1 or 2, recruited between March 2010-April 2012 at the ED of 1 hospital. | **ESS** (Proposed Ensemble-Based Scoring System: combines HRV and 12-lead ECG parameters and vital signs). (Excluded because not routine measurement) **DIST** (an Euclidean distance-based scoring system): uses HRV combined with vital signs (Excluded because not routine measurement) **MEWS** (modified early warning score: reference to Subbe et al 2003) **TIMI** (thrombolysis in myocardial infarction: reference to Antman et al 2000) (Excluded because condition (MI) specific score) | Composite of four severe complications within 72 h of arrival at the ED: mortality, cardiac arrest, sustained ventricular tachycardia, and hypotension requiring inotropes or intraaortic balloon pump insertion. | 19 (3.4%) out of the remaining 564 patients met the composite outcome.  **MEWS** Cut-off: 1.0 AUROC: 0.67 (0.54-0.81) Sensitivity: 42.1% (19.9%-64.3%) Specificity: 78.5% (75.1%-82.0%) PPV: 6.4% (2.1%-10.7%) NPV: 97.5% (96.0%-99.9%) |
| Wilson et al (2016),^61^ UK  **Risk of bias**: High | To evaluate the ability of a data-fusion Patient Status Index (PSI) to detect patient deterioration in the ED in comparison with documented TTS and retrospectively calculated TTS. | Prospective cohort (Validation) | 472 adults (≥18) entering one of three clinical areas of the ED (resuscitation room, ‘majors’, observation ward); during times the research team was available (daytime). | **Vital sign and TTS data**: heart rate (HR), systolic and diastolic blood pressure (BP), respiratory rate, peripheral oxygen saturation (SpO_2_), temperature and Glasgow Coma Scale (GCS) score. **Paper TTS**: clinician assigns score **eTTS**: For each set of manually recorded vital signs, TTS was retrospectively calculated **PSI**: reference to Tarassenko et al (2006); parameters: HR, respiratory rate, BP, temperature, oxygen saturation | Escalation of care | **PSI true alerts** Escalation after ED arrival: 35, of which 20 had PSI data, of which:   - Detected by TTS: 4 - Detected by eTTS: 17 - Detected by PSI: 15 - Detected by eTTS, not PSI: 5 - Detected by PSI, not eTTS: 3   **PSI false alerts** False alert rate: 1.13 alerts/bed-day (49 false alerts from 39 patients). |

***Table 8. Evidence table: Development and validation studies – Patient groups differentiated by (suspected) condition***

| **Authors (year), country** | **Study aim** | **Study design (type)** | **Participants** | **Content of system/tool** | **Reference criteria** | **Results** |
| --- | --- | --- | --- | --- | --- | --- |
| Albright et al. (2014),^29^ USA  **Risk of bias**: Low | To design an emergency department sepsis scoring system to identify risk of ICU admission in pregnant & postpartum women. | Retrospective cohort (Development & validation) | 850 pregnant & post partum women with suspected SIRS or sepsis evaluated in ED of a large tertiary care hospital between Feb 2009-May 2011. | **Sepsis in Obstetrics Score (SOS)**: (Excluded because it is a condition (sepsis) specific tool)  **MEWS**  (Parameters not stated in report.)  **REMS**  (Parameters not stated in report.) | **Primary outcome**: ICU admission within 48 hrs of ED presentation.  **Secondary outcomes:** • telemetry unit admission • length of hospital stay • mortality • positive blood cultures • positive influenza swab • antibiotic use • adverse perinatal outcome | 9 (1.1%) admitted to ICU, 32 (3.8%) to telemetry unit, mortality (0.0%).   **Primary outcome: ICU Admission prediction** MEWS (cut-off ≥5): Sensitivity: 100.0%, Specificity: 77.6%, PPV: 4.6%, NPV: 100.0%  REMS (cut-off ≥6): Sensitivity: 77.8%, Specificity: 93.3%, PPV: 11.1%, NPV: 99.7%   **Secondary outcomes** Not reported for MEWS and REMS |
| Cildir et al (2013),^38^ Turkey  **Risk of bias:** Low | To evaluate the modified Mortality in Emergency Department Sepsis (MEDS) score, MEWS score and CCI to predict prognosis in patients presenting to ED diagnosed with sepsis. | Prospective cohort (Validation) | 230 patients ≥18 who presented to the ED of 1 hospital between 7 Aug 2009-15 Feb 2011 diagnosed with community acquired sepsis. | **Charlson Comorbidity Index (CCI)**: parameters not stated in report **MEWS:** parameters not stated in report **Mortality in emergency department sepsis (MEDS)**: excluded from review because it is condition (sepsis) specific | Mortality | **Prediction of the mortality in the group with sepsis according to 28-day mortality (n = 64) (8 patients died)** CCI (cut-off >5) Sensitivity 50%, Specificity 85.4%, PPV 33.3, NPV 92.2, AUC 0.65 (p=0.18) MEWS (cut-off ≤5) Sensitivity 87.5%, Specificity 30.4%, PPV 15.2, NPV 94.4, AUC 0.57 (p=0.48)  **Prediction of the mortality in the group with severe sepsis according to 28-day mortality (n=166) (66 patients died)** CCI (cut-off >5) Sensitivity 78.8%, Specificity 38%, PPV 45.6, NPV 73.1, AUC 0.62 (p=0.006) MEWS (cut-off ≤5) Sensitivity 48.5%, Specificity 67.0%, PPV 49.2, NPV 66.3, AUROC 0.60 (p=0.04)  **Predictive value of the scores for 28-day mortality**  CCI (cut-off >5) Sensitivity 27.0%, Specificity 93.0%, PPV 64.5, NPV 72.9, AUROC 0.65 (p=0.001) MEWS (cut-off ≤5) Sensitivity 43.2%, Specificity 75.0%, PPV 45.1, NPV 73.6, AUROC 0.61 (p=0.008) |
| Considine et al (2015),^39^ Australia | To evaluate the effect of the staged implementation of a Rapid Response System on reporting of clinical deterioration in ED patients. A secondary aim was to determine if there were differences between patients who did, and did not, experience clinical deterioration documented during ED care. | Retrospective cross-sectional design (Validation) | Stratified random sample of 600 adult ED patients (≥18 years) with presenting with shortness of breath, chest pain or abdominal pain in a 300-bed urban hospital. 150 patients in each of 4 groups. Four groups by stage of implementation: (T0) Clinical decision making/discretion, no TTS chart (year 2009); (T1) Escalation of care protocol (if any critical instability criteria met, an ED physician should review within 5min), no TTS chart (year 2010); (T2) Escalation of care protocol, single parameter TTS chart (year 2011); (T3) Escalation of care protocol, single parameter TTS chart (year 2012). | **Critical instability criteria (ED CIC):**  • Airway/ breathing: Stridor, upper airway obstruction, or threatened airway, SpO2 < 90% (on oxygen 10 L/min via mask), Arterial blood gases pH < 7.20, Respiratory rate < 10 breaths/min or > 30 breaths/min  • Circulation: Heart rate < 50 beats/min or > 120 beats/min, Systolic blood pressure < 90 mmHg or > 200 mmHg, Urine output < 20 mL/h or < 100 mL/6 h  • Disability: Sudden decrease in consciousness (fall in Glasgow Coma Scale score > 2), Repeated or prolonged seizures  • Worried?: Patients who may not meet the above criteria but have a sudden deterioration in their medical condition, requiring urgent medical review. | **Primary outcome**:  Unreported clinical deterioration (=presence of documented physiological abnormalities that fulfilled the ED Clinical Instability Criteria in ED nursing notes and no documentation that these were reported to a medical officer.) | At T0, 86.7% of episodes of clinical deterioration were unreported. Across the four years studied, episodes of unreported clinical deterioration decreased by 17.9% from T0 to T1 (68.8%), 13.5% from T1 to T2 (55.3%) but only 1.3% from T2 to T3 (54.0%); none of these differences were statistically significant (p = 0.14).  Patients who experienced clinical deterioration in the ED were more likely to arrive by ambulance (p < 0.001), be triaged to Australian Triage Scale categories 1 or 2, (p < 0.001), and had a 2.8 hour longer median ED length of stay, and were 31.9% more likely to be admitted to hospital (p < 0.001). |
| Corfield et al (2014)^74^ (and related conference abstract Corfield et al (2012),^68^ Scotland  **Risk of bia**s: Low | To determine whether a single National Early Warning Score (NEWS) on ED arrival is a predictor of outcome in patients with sepsis, either in-hospital death in 30 days or ICU admission within 2 days | Retrospective cohort (Validation) | 3890 (74% of 5285 eligible patients) adult patients, >16 years of age, attending ED with sepsis (suspected or confirmed within 2 days of attendance and 2 or more of sepsis criteria). 20/25 Scottish mainland EDs participated. | NEWS (0-20 score) Parameters: respiratory rate, oxygen saturations, temperature, SBP, pulse, conscious level, supplemental O_2_ | ICU admission within 2 days of attendance at ED and 30-day mortality (in-hospital). A combined endpoint of ICU admission/and or mortality was also assessed. | Included in analysis: n = 2003  ICU (within 2 days): n = 113 (6.0%)  30-day mortality: n = 297 (15.0%)  Combined (ICU and/or mortality): n = 376 (19.0%)   **ICU (within 2 days)** (Compared to NEWS 0-4; Adjusted for age) NEWS Score  5-6: OR 1.22 (95% CI 0.59-2.54; p=0.59) 7-8: OR 2.01 (95% CI 1.02-3.97; p=0.04)  9-20: OR 5.76 (95% CI 3.22-10.31; p=0.00)  **Mortality (30 days)** (Compared to NEWS 0-4; Adjusted for age) NEWS Score  5-6: OR 1.95 (95% CI 1.21-3.14; p=0.01)  7-8: OR 2.26 (95% CI 1.42-3.61; p=0.004) 9-20: OR 5.64 (95% CI 3.70-8.60; p=0.00)  **Combined (ICU and/or mortality)** (Compared to NEWS 0-4; Adjusted for age) NEWS Score  5-6: OR 1.72 (95% CI 1.14-2.60; p=0.01)  7-8: OR 2.17 (95% CI 1.45-3.25; p=0.00) 9-20: OR 5.78 (95% CI 4.02-8.31; p=0.00)   Cut-off point with highest Youden’s Index: NEWS 9: Sensitivity 0.52, specificity 0.77, PPV 0.35, NPV 0.88, Youden’s index 0.30 |
| Geier et al (2013),^32^ Germany  **Risk of bias**: Low | (1) To evaluate Emergency Severity Index (ESI), Modified Early warning Score (MEWS), Mortality in Emergency Department Sepsis (MEDS) score concerning their diagnostic accuracy to detect patients with Severe Sepsis and Septic Shock score (SSSS).  (2) To determine the prognostic accuracy of indices in predicting the in-hospital mortality of patients with suspected sepsis in ED.  (3) To calculate the prognostic value of the Charlson Comorbidity Index (CCI). | Prospective cohort (Development & Validation) | 151 consecutive adult patients with suspected sepsis admitted to the ED of 1 hospital between 1 Aug-30 Sept 2012. | **Emergency Severity Index (ESI)** (5 levels; the higher the level the lower the medical urgency) Level 1 = acute life threatened ill patients - require immediate initiation of diagnostics and therapy.  Level 2 = patients in high-risk situation - initiation of diagnostics and therapy has to start within 10min following the initial triage assessment.  Higher levels not specified in report.  **MEWS** (0-14)  Parameters: SBP, HR, Temperature, respiratory rate, LOC   **CCI Score** (0-37) Parameters not specified in report.  **MEDS score**  (Excluded because it is a condition (sepsis) specific score). | In-hospital mortality | 45.0% (n=72) diagnosed with SSSS; 33.1% (n=53) uncomplicated sepsis (without organ dysfunction). 21.9% (n=26) no sepsis, but SIRS or locally confined infection.  **In-hospital mortality** (14.6% of all patients; 27.8% of patients with SSSS)  ESI  Sensitivity 0.73, Specificity 0.0, PPV 0.17, NPV 0.90 MEWS  Sensitivity 0.43, Specificity 0.74, PPV 0.21, NPV 0.89 CCI  Sensitivity 0.82, Specificity 0.64, PPV 0.21, NPV 0.94 |
| Howell et al (2007),^45^ USA  **Risk of bias**: Low | To validate the Mortality in Emergency Department Sepsis (MEDS) score, the Confusion, Urea nitrogen, Respiratory rate, Blood pressure, 65 years of age and older (CURB-65) score, and a modified Rapid Emergency Medicine Score (mREMS) in patients with suspected infection. | Prospective cohort (Validation) | 2132 adult patients with clinically suspected infection admitted to an urban ED between 10 Dec 2003 - 30 Sept 2004. | **mREMS** points depend on severity Mean arterial pressure (mm Hg) Pulse rate Respiratory rate Peripheral oxygen saturation Glasgow Coma Score  Age   **MEDS score** (Excluded because it is a condition (sepsis) specific score.)  **CURB-65 score** (Excluded because it is a condition (community-acquired pneumonia) specific score.) | 28-day in-hospital survival (patients discharged alive from the hospital before 28 days were considered alive for the 28-day in-hospital mortality end point). | Of 2,132 patients with unique first visits, 83 (3.9%; 95% CI 3.1% to 4.7%) died.   **mREMS**  Odds of death increased by 1.40 (95% CI = 1.28 to 1.45) with each point increase. AUROC 0.80 (0.75-0.85) |
| Jo et al (2013),^46^ Korea  **Risk of bias**: Low | To compare the predictive value of the VitalPAC Early Warning Score-Lactate (VIEWS-L) score with that of the Trauma Injury Severity Score (TRISS). | Retrospective cohort (Validation) | 299 patients, ≥ 15 years of age, with blunt trauma, Injury severity score ≥9 in a 1000-bed urban hospital between 1 Apr 2010-31 March 2011. | **VIEWS-L**  Parameters: SBP, HR, respiratory rate, temperature, oxygen saturations, inspired oxygen, central nervous system alertness  **TRISS**  (Excluded because it is a trauma-specific tool) | In-hospital mortality | **VIEWS-L**  Higher score in non-survivors (median 7.9, IQR 6.2-12.9) than non-survivors (median 3.7, IQR 1.7-5.5).  AUROC: 0.83 (95% CI 0.77-0.91) |
| Jo et al. (2016),^47^ Korea  **Risk of bias**: Low | To investigate the prognostic prediction power of newly introduced early warning score modified by serum lactate level, the National Early Warning Score (UK) including Lactate (NEWS-L), among community-acquired pneumonia patients, and compared with previously used tools such as Pneumonia Severity Index and CURB-65. | Retrospective cohort (Validation) | 553 patients, ≥18 years of age, with an admission diagnosis of any type of pneumonia between 1 Oct 2013-30 Sept 2014 in 1 hospital. | **NEWS-L score** Parameters: SBP, HR, respiratory rate, temperature, SpO_2_, LOC, supplemental oxygen, lactate level  **CURB-65** (Excluded because it is a condition (community-acquired pneumonia) specific tool)  **Pneumonia Severity Index**  (Excluded because it is a condition (pneumonia) specific tool) | Inpatient mortality | Mortality by NEWS-L score:  ≤ 3.0: 2.2%  3.1 ≤ and ≤ 5.2: 7.9% 5.3 ≤ and ≤ 8.0: 9.6% ≥8.1: 23.9%   **NEWS-L**  AUROC; 0.73 (0.66-0.80); reference  Cut-off ≥3.1: Sensitivity 95.0 (86.1-99.0) Specificity 27.6 (23.7-31.8) PPV (%) 13.8 (10.6-17.5) NPV (%) 97.8 (93.8-99.6) PLR 1.3 (1.2-1.4) NLR: 0.2 (0.06-0.6) Cut-off ≥5.3: Sensitivity 76.7 (64.0-86.6) Specificity 53.8 (49.2-58.2) PPV 16.8 (12.6-21.8) NPV 95.0 (91.7-97.2) PLR 1.7 (1.4-2.0) NLR 0.4 (0.3-0.7) Cut-off ≥8.1: Sensitivity 55.0 (41.6-64.9), Specificity 78.7 (74.8-82.2), PPV 23.9 (17.1-31.9), NPV 93.5 (90.7-95.7), PLR 2.6 (1.9-3.4), NLR 0.6 (0.4-0.8) Cut-off ≥7.3: Sensitivity 63.3 (49.9-75.4), Specificity 73.2 (69.1-77.1), PPV 22.4 (16.3-29.4), NPV 94.3 (91.4-96.4), PLR 2.4 (1.9-3.0), NLR 0.5 (0.4-0.7)  **NEWS**  AUROC 0.70 (0.63-0.77); p=0.03  Cut-off ≥5 including red score: Sensitivity 68.3 (54.9-79.4), Specificity 57.2 (52.7-61.6), PPV 16.3 (12.1-21.5), NPV 93.7 (90.2-96.1), PLR 1.60 (1.3-2.0), NLR 0.55 (0.4-0.8) |
| Jones et al (2005),^48^ USA  **Risk of bias**: Low | The hypothesis of this study states that the physiologic scoring systems New SimplifiedAcute Physiology Score II (SAPS II), Morbidity Probability Model  (MPM0 II), and Logistic Organ Dysfunction System (LODS), when calculated from variables available during the care of critically ill ED patients, will not perform with high diagnostic accuracy for predicting hospital mortality, defined as an area under the receiver operating characteristic (ROC) curve <0.80. | Secondary analysis of a randomized controlled trial (Validation) | 91 (45% of eligible patients) non-trauma ED patients admitted to an intensive care unit, >17 years of age, with initial ED vital signs consistent with shock (systolic blood pressure <100 mm Hg or shock index >1.0), and with agreement of two independent observers for at least one sign and symptom of inadequate tissue perfusion. | **SAPS II** **MPM0 II LODS** No data provided on included parameters. | In-hospital mortality | The in-hospital mortality rate was 21% (19/91). **SAPS II** Mean 40 (SD 14)  Predicted mortality 28% AUROC 0.72 (0.57-0.87)  **MPM0 II** Mean -1.06 (SD 1.24)  Predicted mortality 28% AUROC 0.69 (0.54-0.84)  **LODS** Mean 5 (SD 3)  Predicted mortality 30% AUROC 0.60 (0.45-0.76)  Using only ED variables to calculate the scores resulted in all three scoring systems overestimating in-hospital mortality by a mean of 8% (range, 7–10%).  The scoring systems appear to function most accurately in the lower risk group for each scoring system (SAPS II ≤50, LOD ≤7, and MPM0 II ≤0) with an average difference between actual and predicted mortality of only 3%. All three of the scoring systems greatly overestimated mortality in the higher risk group, with an average difference between actual and predicted mortality of 31%. |
| Nguyen et al (2012),^59^ USA  **Risk of bias:** Unclear | To examine the performance of the Predisposition, Insult/Infection, Response, and Organ dysfunction (PIRO) model compared with the Acute Physiology and Chronic Health Evaluation (APACHE) II and Mortality in Emergency Department Sepsis (MEDS) scoring systems in predicting in-hospital mortality for patients presenting to the ED with severe sepsis or septic shock. | Prospective cohort (Validation) | 541 patients, >17 years of age, who met the criteria for high-R severe sepsis (sepsis with lactate ≥4 mmol/L) or septic shock in the ED. | **PIRO**  Parameters: Age, chronic liver disease, and/or congestive cardiomyopathy Infection  Tachycardia/tachypnoea Organ dysfunction  **APACHE II** reference Knaus et al (1985)  **MEDS** (Mortality in Emergency Department Sepsis; reference Sapiro et al 2003) (Excluded because it is a condition (sepsis) specific tool) | In-hospital mortality | 62% (61.9) of patients were diagnosed with septic shock; 63.4%, with positive culture; and 46.9%, with positive blood culture. During the course of care, 31.8% patients died in the hospital.  **PIRO** Predicted mortality: 48.5% (40.1 and 63.9)  AUROC: 0.71 (0.66-0.75)  **APACHE II**  Predicted mortality: 66% (42 and 83) AUROC: 0.71 (0.66-0.76)  Actual mortality significantly increased with increasing PIRO score in patients with either APACHE II less than 25 (P=0.01) or APACHE II 25 or more (P<0.01). The PIRO consistently overestimated actual mortality when stratified by levels of predicted mortality. |
| Vorwerk et al (2009),^51^ UK  **Risk of bias:** Low | To determine the efficacy of the abbreviated Mortality in Emergency Department Sepsis (MEDS) score (without neutrophil bands), the MEWS score and NPT lactate in predicting 28-day mortality in adult ED patients with sepsis. | Retrospective cohort (Validation) | 307 adult ED patients (>16 years) with sepsis admitted to 2 hospitals. Patients were excluded if parameters for MEW or MEDS score were missing. Mean age 69.7 years (95% CI 67.5 to 71.8); 51% men. | **MEWS:** 5 Parameters: SBP, pulse rate, respiratory rate, temperature, AVPU score **Abbreviated MEDS** (Excluded because it is a condition (sepsis) specific score) **Blood lactate**  Was only routinely measured in the ED of 1 of the hospitals (n = 158) | 28-day mortality | **MEWS** MEWS ≥5  Sensitivity: 72.2% (95% CI 60.4% to 82.1%)  Specificity: 59.2% (95% CI 52.6% to 65.5%) Significant predictor for non-survival (OR 3.76; 95% CI 2.11 to 6.71). AUROC: 0.72 (0.67 to 0.77)  **Lactate** Lactate level of ≥4 mmol/l Sensitivity: 49.1% (95% CI 35.1% to 63.2%) Specificity: 74.3% (95% CI 64.8% to 82.3%) Significant predictor for non-survival (OR 2.80; 95% CI 1.39 to 5.57).  AUROC: 0.62 (0.54 to 0.70) |
| Williams et al. (2016),^52^ Australia  **Risk of bias:** Low | (1) To validate a number of severity of illness scores in patients admitted with presumed infection;  (2) To compare the performance of scores in patient subgroups with increasing mortality: infection without systemic inflammatory response syndrome, sepsis, severe sepsis and septic shock | Prospective cohort (Validation) | 8,871 patients admitted with presumed infection, >17 years of age, to ED of 1 metropolitan hospital in 160 weeks in 2 periods: Oct 2007-Dec 2008 (pilot) and June 2009-May 2011 (funded). | **Simplified Acute Physiology Score (SAPS II)** Parameters: Age, chronic disease, type of admission, GCS, temp, BP, HR, FiO_2_/PaO_2_/Aa gradient, bicarbonate, sodium, potassium, WBC, bilirubin, urea, urine output. **Sequential Organ Failure Assessment (SOFA)** Parameters: GCS, BP, vasopressor use, FiO_2_/PaO_2_/Aa gradient, platelets, bilirubin, creatine, urine output. **Acute Physiology and Chronic Health Evaluation II (APACHE II) score** Parameters: Age, chronic disease, type of admission, GCS, temp, BP, HR, FiO2/PaO2/Aa gradient, pH, respiratory rate, sodium, potassium, WBC, haematocrit, creatinine, renal failure. **Severe Sepsis Score (SSS)** (Excluded because it is a condition (sepsis) specific score) **Mortality in ED Sepsis Score (MEDS)** (Excluded because it is a condition (sepsis) specific score) | 30-day mortality | 30-day mortality: 3.7%. All scores overestimated mortality. Scores in ICU settings overestimated mortality in ED.  **AUROC (95% CIs) for Scores, by Sepsis Subgroups**   APACHE II  Entire Cohort = 0.90 (0.88–0.91) Sepsis: 0.86 (0.84–0.88)  Severe Sepsis: 0.79 (0.76–0.83) Septic Shock: 0.79 (0.74–0.84) ICU: 0.77 (0.67–0.87)  SAPS II Entire Cohort: 0.90 (0.89–0.92) Sepsis: 0.88 (0.86–0.90) Severe Sepsis: 0.82 (0.78–0.85) Septic Shock: 0.82 (0.78–0.87) ICU: 0.80 (0.71–0.88)  SOFA Entire Cohort: 0.86 (0.84–0.88) Sepsis: 0.83 (0.80–0.86) Severe Sepsis: 0.78 (0.75–0.82) Septic Shock: 0.74 (0.68–0.79) ICU: 0.74 (0.65–0.84) |

***Table 9. Evidence table: Development and validation studies – Undifferentiated patient groups***

| **Authors (year), country** | **Study aim** | **Study design (type)** | **Participants** | **Content of system/tool** | **Reference criteria** | **Results** |
| --- | --- | --- | --- | --- | --- | --- |
| Burch et al (2008),^63^ South Africa  **Risk of bias**: High | To evaluate the use of the Modified Early Warning Score (MEWS) as a triage tool to identify medical patients presenting to the emergency department who require admission to hospital and are at increased risk of in-hospital death. | Prospective cohort (Validation) | 790 (70.2% of the potential study cohort) medical patients (not surgical, orthopaedic, gynaecological or trauma related) presenting to the ED of an urban public hospital. | **MEWS:** Parameters: SBP, pulse rate, respiratory rate, temperature, AVPU score | Admission to hospital, in-hospital mortality | MEWS score median 3 (range 0-11); 26.0% had MEWS score ≥ 5.    **Hospital admission** Increased admission with increasing MEWS (trend P-value <0.001). MEWS 0–2 (45%; ref)  MEWS 3–4 (59%; RR 1.3; 95% CI 1.1 to 1.6)  MEWS ≥5 (79%; RR 1.7; 95% CI 1.5 to 2.0)   **In-hospital mortality** Increased mortality with increasing MEWS (trend P-value <0.001). MEWS 0–2 (5%; ref)  MEWS 3–4 (16%; RR 2.8; 95% CI 1.7 to 4.8)  MEWS ≥5 (26%; RR 4.6; 95% CI 2.7 to 7.8) |
| Correia et al (2014),^55^ Portugal  **Risk of bias**: Unclear | To assess the Early Warning Score (EWS) in specific time windows preceding an acute event, to study its temporal behaviour and its relation to outcomes, to compare it with established ward care. | Retrospective cohort | First consecutive 100 adult ward patients assisted by the outreach team and transferred to ED from 1 Jan to 31 April 2009. | EWS: parameters not clearly specified. Score threshold of >3 as trigger. | Length of hospital stay, mortality | n=65 (65% of eligible sample)  • Main cause of deterioration: Respiratory problems (44.6%); cardiovascular (27.7%) and neurological deterioration (27.7%). • EWS score at three periods preceding ward transfer to the ED (EWS Mean/SD):  72 h: 2.6 ± 1.9   24 h: 2.4 ± 1.8   12 h: 3.8 ± 1.7  • Score at 24h and 12h seemed to predict both length of stay and mortality (p < 0.05).  • 63% were admitted in ICU or Intermediate Care Units* (26% and 37%, respectively), 20% returned to their origin wards, and 17% died in the ED. The overall in-hospital mortality = 53.8% • The EWS would have increased early medical attention by 40% if a threshold of ≥3 was used.  * This study describes 3 levels of care (return to ward, admission to Intensive or Intermediate Units) |
| Dundar et al (2015),^41^  Turkey  **Risk of bias:** Low | The aim of this study was to evaluate the value of the Modified Early Warning Score (MEWS) and the VitalPac Early Warning Score (VIEWS) in predicting hospitalization and in-hospital mortality in geriatric emergency department (ED) patients. | Prospective cohort (Validation) | 671 (all) patients (aged ≥65 years) presenting to the ED of 1260-bed hospital between 15 January 2014 and 15 February 2014. | **MEWS**  Parameters: respiratory rate, SBP, HR, Temp, AVPU  **VIEWS**  Parameters: respiratory rate, SBP, HR, Temp, oxygen saturation, inhaled oxygen, AVPU | Hospitalization & in-hospital mortality | 187 (27.9%) admitted to a ward  153 (22.8%) were admitted to ICU  4 (0.6%) patients died during the follow-up at the ED  8.5% in-hospital mortality rate  **Hospitalisation**  MEWS (optimal cut-off: 3)  AUROC: 0.73 (95% CI 0.69–0.77)  Sensitivity: 42%, Specificity: 89%  LR+: 3.7, LR−: 0.7  VIEWS (optimal cut-off: 6)  AUROC: 0.76 (95% CI 0.72–0.79)  Sensitivity: 56%, Specificity: 85%  LR+: 3.8, LR−: 0.5.  **In-hospital mortality**  MEWS (optimal cut-off ≥4)  AUROC: 0.89 (95% CI 0.84–0.94)  Sensitivity: 74%, Specificity: 89%  LR+: 6.7, LR−: 0.3  VIEWS (optimal cut-off: ≥8)  AUROC: 0.90 (95% CI 0.86–0.94)  Sensitivity: 84%, Specificity: 83%  LR+: 4.9, LR−: 0.2.  There was no statistical difference between AUROC of MEWS & VIEWS in predicting hospitalization and in-hospital mortality (P=0.28 and 0.82). |
| Eick et al (2015),^42^ Germany  **Risk of bias**: Low | To evaluate heart rate deceleration capacity, an electrocardiogram-based marker of autonomic nervous system activity, as risk predictor in a medical emergency department and to test its incremental predictive value to the Modified Early Warning Score (MEWS). | Prospective cohort (Validation) | 5730 consecutive patients ≥18 years admitted to the ED of 1 hospital over a 22 month period and in sinus rhythm. | **MEWS**  Parameters: respiratory rate, SBP, HR, Temp, LOC at ED admission   **Heart rate variability (deceleration capacity)** (excluded because not routine assessment) | **Primary outcome**: intra-hospital mortality  **Secondary outcome**: total mortality at 30 and 180 days, transfer to ICU during hospital stay | Admission to ICU (%) n=366 (6.4%) In-hospital deaths: n=142 (2.5%) Deaths at 30 days: n=196 (3.4%) Deaths at 180 days: n=436 (7.6%)  Mean (SD) MEWS Survivors 2.3±1.4 vs Non-survivors MEWS= 5.0±1.7 (p< 0.001)    **In-hospital mortality:** MEWS:  AUROC: 0.71 (0.67–0.75; p< 0.001) Adjusted OR 1.14 (95% CI 1.09–1.19)  **Secondary outcomes** not reported for MEWS. |
| Graham et al (2007),^56^  Hong Kong  **Risk of bias:** Unclear (Conference abstract only) | To validate the use of a Modified Early Warning Score (MEWS) in ED to identify patients at risk of serious illness requiring hospital admission. | Prospective cohort (Validation) | 413 patients (96.5% of eligible patients) admitted to a 16-bed ED observation ward of 1 hospital. | **MEWS**  Parameters: respiratory rate, SBP, HR, Temp, LOC | Inpatient hospital admission, reattendance at ED within 48 hrs related to index of diagnosis after discharge, 30 day mortality. | Admitted: 46 Reattended within 48 hrs of discharge (4 required admission): 10  Deceased 30 days: 2   MEWS score >4: • Increased need for hospital admission OR 8.3: 95% CI = 1.1-60.4, p=0.013)  • Increased ED reattendance within 48 hrs (OR 45.2: 95% CI=3.4 to 568.9, p< 0.0001) (Limited information; abstract only) |
| Heitz et al (2010),^43^ USA  **Risk of bias**: Low | Examine the performance characteristics and discriminatory ability of the most abnormal Modified Early Warning Score (MEWS) (MEWS Max) score during the entire ED stay in predicting the need for higher levels of care among ED patients presenting to a tertiary care facility. | Retrospective cohort (Validation) | 280 of 500 randomly selected charts of all patients presenting to the ED of one Medical Centre in 2005. | **Adapted MEWS (MEWS Max)**: Parameters: SBP, pulse rate, respiratory rate, temperature, Glasgow Coma Scale (GSC). **MEWS plus**: Parameters: MEWS max, age 60, race, gender, ED length of stay, method of arrival, and antibiotics given prior to or during ED visit. | Composite of: Need for higher level of care (defined as initial admission from the ED or transfer within 24 hours to a non-floor bed (acute care, intermediate care unit, or critical care unit) or mortality within 24 hours of ED presentation. | 27% (76/280) met composite outcome of death (n=1) or need for higher care (n=75).  **MEWS Max** The MEWS Max was significantly associated with the primary composite outcome (P < 0.001, Cochran-Armitage trend test).  Optimum threshold MEWS Max: ≥4  Sensitivity: 62% (95% CI 50-73) Specificity: 79% (95% CI 73-84) PPV: 52 NPV: 85 AUROC 0.73 (95% CI, 0.66- 0.79) Each 1-point increase in the MEWS Max score associated with a 60% increase in the odds of meeting the composite endpoint (OR 1.6; 95% CI, 1.3-1.8).  **MEWS Plus**  AUROC 0.76 (95% CI, 0.69- 0.82) In 58 cases (21.7%), using MEWS Plus would have placed patients in a more appropriate risk category than MEWS Max, while 5.6% of cases would have resulted in inappropriate reclassification. |
| Junhasavasdiku et al (2012),^58^ Thailand  **Risk of bias:** Unclear | To determine whether admission delay (lead-time) and other factors are associated with hospital mortality rates of emergency medical patients. (Modified Early Warning Score (MEWS) data used in this synthesis). | Prospective cohort (Validation) | 381 patients, >15 years of age, presenting to the ED between Aug-Nov 2009 and admitted to medical wards of a tertiary urban care centre, including intensive care units. | **MEWS:** Parameters: SBP, pulse rate, respiratory rate, temperature, AVPU score | Mortality | Overall mortality rate was 8.9%. MEWS at ED was associated with mortality (p<0.001): Non-survivors median 4 (range 1-10), survivors median 2 (range 0-11) |
| Naidoo et al (2014),^62^ South Africa  **Risk of bias:** High | To evaluate the use of the Triage Early Warning Score (TEWS) by healthcare workers in an ED in a large urban hospital in KwaZulu-Natal, and its ability to identify patients who require admission and at increased risk for in-hospital mortality. | Retrospective cohort (Validation) | 265 patient records in an ED of 1 urban hospital. | **TEWS** :  Parameters: Mobility, Resting rate, HR, SBP, AVPU, Trauma | Discharge within 24 hours of admission, admission to a ward, admission to an intensive care unit (ICU), and death in hospital. | 47.6% were admitted to wards and 3 (1.1%) admitted to ICU; 4 patients (1.5%) died within 24 hours of admission. 233 (87.9%) had a TEWS < 7, while 32 (12.1%) had a TEWS ≥ 7.  A significant association between the TEWS category and outcome was established (no details of significance tests provided):  53.7% of patients with a TEWS of < 7 were discharged, compared to 18.7 % with a score ≥ 7 who were discharged. No patients in the low-score category were admitted to ICU. No patients died. Three patients were admitted to ICU, and four died in the high-score category. |
| Olsson et al (2003),^33^ Sweden  **Risk of bias:** Low | (1) Could the abbreviated severity of disease classification system Rapid Acute Physiology Score (RAPS), created for use in the out-of-hospital setting, be useful in the ED for predicting in-hospital mortality and the length of hospital stay (LOS) in nonsurgical patients? (2) Is it possible to modify RAPS to provide a more potent scoring system (the Rapid Emergency Medicine Score [REMS]) by including age and one or two parameters easily obtained by modern technology (oxygenation and body temperature) for the purpose of predicting in-hospital mortality? (3) Could REMS, with its simplicity and fewer variables, perform as well as APACHE II in the nonsurgical ED? | Prospective cohort (Development & Validation) | 1027 adult nonsurgical patients were recruited from two sources: 185 nonsurgical, critically ill patients referred to the ICU from 1 Nov 1995 -1 Nov 1996, and 885 patients at the nonsurgical ED who were admitted either to an ordinary medical department (n = 758), to a general ICU (n = 9), to a coronary care unit (n = 84), or to a neuro-ICU (n = 15) between 1 Jan 1996 -1 March 1996. | **Model Validation** **APACHE II** Parameters: temperature, mean arterial pressure, HR, oxygenation of arterial blood (PaO_2_), arterial pH, serum sodium, serum potassium, serum creatinine, haematocrit, white blood cell count, and GCS score. (Arterial pH was not used in the scoring system because this variable is not measured routinely in the ED.)  **RAPS** Parameters: HR, BP, respiratory rate, and GCS score.  + peripheral oxygen saturation (0–4 points), body temperature (0–4) and age were added to the four RAPS variables. **Model development**  **REMS** (based on best predictors of RAPS) Parameters: coma, respiratory frequency, oxygen saturation, BP, and HR (maximal score being 4 for all) and age (maximal score being 6). | In-hospital mortality | Mortality of 116 (11%).  **REMS** Likelihood ratio chi-square value of 318.7 (p < 0.0001)  OR 1.58 (95% CI 1.48 to 1.70). AUROC: 0.91 +/- 0.02 (had a superior discriminating power compared to RAPS (p<0.001))  **RAPS** Likelihood ratio chi-square value of 273 (p<0.0001) OR 1.77 (95% CI 1.62 to 1.93). AUROC: 0.87 +/- 0.02  **APACHE II** Likelihood ratio chi-square value of 278.5 (p <0.0001)  OR 1.25 (95% CI 1.21 to 1.29). AUROC: 0.90 +/- 0.02 (no significant difference with REMS) |
| Olsson et al (2004),^34^  Sweden  **Risk of bias:** Low | (1) Could the abbreviated severity of disease classification system Rapid Acute Physiology Score (RAPS), created to be used in the pre-hospital setting, be useful in the ED to predict in-hospital mortality and hospital length of stay (LOS) in nonsurgical patients?; (2) Is it possible to modify RAPS to provide a more powerful scoring system for medical patients (Rapid Emergency Medicine Score, REMS) to predict in-hospital mortality? | Prospective cohort (Development & Validation) | 11751 nonsurgical patients presenting to the ED during 12 consecutive months. | **RAPS**  Parameters: blood pressure, respiratory rate, pulse rate and Glasgow coma scale  Model Developed **REMS** (based on significant predictors of RAPS) Parameters: coma, respiratory frequency, oxygen saturation, blood pressure and pulse rate (maximal score being 4 for all) and age (maximal score being 6) | In-hospital mortality | Mortality: n=285  **RAPS** Likelihood ratio chi-square value of 261.2 (P < 0.0001)  OR 1.47 (95% CI: 1.41–1.54). AUROC: 0.65 ± 0.02  **REMS** Likelihood ratio chi-square value of 487.3 (P < 0.0001)  OR 1.40 (95% CI: 1.36–1.45) AUROC: 0.85 ± 0.01; superior discriminating power compared with RAPS (P < 0.001) |
| Subbe et al (2006),^50^ UK  **Risk of bias:** Low | To establish a frequency distribution for typical physiological scoring systems and to establish the potential benefit of adding these to an existing triage system in accident and emergency departments. | Retrospective cohort (Validation) | Group 1: 53 unselected patients presenting at ED in two samples of consecutive patients on 30 and 31 Oct 2003.  Group 2: 49 direct admissions from ED to the ICU admitted between 1 April-31 Oct 2003.  Group 3: 49 patients admitted to ED, who were transferred to a general medical or surgical ward and then admitted to ICU between 1 April-31 Oct 2003.  Total of 151 patients. | **MEWS** (Modified Early Warning Score)**:** Parameters: SBP, pulse rate, respiratory rate, temperature, AVPU score **ASSIST** (Assessment Score for Sick patient Identification and Step-up in Treatment) Parameters: SBP, pulse rate, respiratory rate, level of consciousness (ACDN score), age.  **MET** (Medical Emergency Team) Criteria for the call-out of a MET based on Airway, Breathing, Circulation, Disability assessment. Nursing staff are asked to call out senior staff if bedside observations are below or above defined thresholds for blood pressure, heart rate, respiratory rate & level of consciousness, or if worried about a patient. Existing triage system**:** **MTS** (Manchester Triage System) Uses protocols based on the presenting complaint and questions about aggravating factors. | Critically ill (defined as MEWS >2, ASSIST >3 and MET criteria applicable, with MTS categories orange or red). | Patients identified as critically ill (at risk of deterioration): **MTS** (orange or red) Group 1: Sensitivity 15% Group 2: Sensitivity 96% Group 3: Sensitivity 65%  **MEWS** (>2) Group 1: Sensitivity 8% Group 2: Sensitivity 77% Group 3: Sensitivity 55%  **ASSIST** (>3) Group 1: Sensitivity 0% Group 2: Sensitivity 22% Group 3: Sensitivity 16%  **MET** (=1) Group 1: 0 Group 2: Sensitivity 2% Group 3: Sensitivity 7% |
| Wang et al (2016),^60^ Taiwan  **Risk of bias:** Unclear | To evaluate whether peri-arrest Modified early Warning Score (MEWS) could be a prognostic factor in in-hospital cardiac arrest (IHCA). To combine pre-arrest comorbidity factors (Charleson Comorbidity Index, CCI), peri-arrest physiological factors (MEWS) and arrest factors to evaluate the outcome of IHCA in ED. | Retrospective cohort (Validation) | 99 non-traumatic, >20 years of age, patients’ ED records of one hospital over 30 months period. | **Charlson Comorbidity Index** 1-6 points allocated by morbidity. **Peri-arrest MEWS** Temp, BP, HR, Respiratory Rate, LoC (AVPU) from triage to 0.5hrs prior to arrest (peri-arrest MEWS) | In-hospital cardiac arrest, Survival to discharge (STDG) | Lower CCI in STDG group (n=22) than mortality group (n=77) (2.27±1.87 vs 3.87±2.83; p=0.001) No significant difference in MEWS at triage between STDG vs mortality group (3.42± 2.2 vs 4.02 ± 2.65; p=0.81) Lower periarrest MEWS in STDG vs mortality group (4.41± 2.28 vs 5.82 ± 2.84; p=0.05)  **Survival to discharge**  CCI  Adjusted OR 0.57 (95% CI 0.38-0.84); p=0.005   Peri-arrest MEWS  Adjusted OR 0.77 (95% CI 0.60-0.97); p=0.028 |
